# The Sense of Smell (SoS) Atlas: Its Creation and First Application to Investigate COVID‐19 Related Anosmia With a Comprehensive Quantitative MRI Protocol

**DOI:** 10.1002/jmri.70128

**Published:** 2025-10-03

**Authors:** Marta Gaviraghi, Eleonora Lupi, Elena Grosso, Andrea Fusari, Mattia Baiguera, Anita Monteverdi, Marco Battiston, Francesco Grussu, Baris Kanber, Ferran Prados Carrasco, Rebecca S. Samson, Janine Makaronidis, Marios C. Yiannakas, Michael S. Zandi, Rachel L. Batterham, Egidio D'Angelo, Fulvia Palesi, Claudia A. M. Gandini Wheeler‐Kingshott

**Affiliations:** ^1^ Department of Brain and Behavioral Sciences University of Pavia Pavia Italy; ^2^ Digital Neuroscience Centre IRCCS Mondino Foundation Pavia Italy; ^3^ NMR Research Unit, Queen Square MS Centre, Department of Neuroinflammation, UCL Queen Square Institute of Neurology, Faculty of Brain Sciences University College London London UK; ^4^ ASG Superconductors s.p.a. Genova Italy; ^5^ Radiomics Group, Vall d'Hebron Institute of Oncology Vall d'Hebron Barcelona Hospital Campus Barcelona Spain; ^6^ Department of Medical Physics and Biomedical Engineering, UCL Hawkes Institute University College London London UK; ^7^ E‐Health Center Universitat Oberta de Catalunya Barcelona Spain; ^8^ Centre for Obesity Research, Department of Medicine University College London London UK; ^9^ National Institute of Health Research UCLH Biomedical Research Centre London UK; ^10^ Department of Diabetes and Metabolism, Royal London Hospital Barts Health NHS Trust London UK; ^11^ Department of Neuroinflammation UCL Queen Square Institute of Neurology London UK

**Keywords:** COVID‐19 anosmia, hindbrain, multisequence‐MRI, neuroinflammation and myelin alterations, sense of smell atlas, tractography

## Abstract

**Background:**

The loss of smell (anosmia) has been noted in numerous diseases, including COVID‐19. Inflammatory and microstructural alterations are possible underlying mechanisms of anosmia in COVID‐19. However, no atlas exists to study olfaction and the associated tissue property changes.

**Purpose:**

To develop the sense of smell (SoS) atlas, including gray matter regions and white matter tracts of the olfactory circuit, to investigate the underpinnings of COVID‐19 related anosmia.

**Study Type:**

Retrospective.

**Subjects:**

For the SoS atlas, high‐resolution tractograms of 10 healthy controls (HC) of the Human Connectome Project (7 females, 22–35 years) were used. The SoS atlas was applied to 8 subjects with persistent anosmia following COVID‐19 (COVID‐P, 7 females, 52 ± 12 years), 19 subjects that recovered from COVID‐19 anosmia (COVID‐R, 14 females, 38 ± 13 years), and 17 HC (8 females, 39 ± 12 years).

**Field Strength/Sequence:**

3 T, 3D inversion recovery, 3D fast field echo, and spin‐echo echo‐planar imaging sequences.

**Assessment:**

To create the SoS atlas, regions were identified and tracts were extracted via tractography following biological constraints. MRI metrics sensitive to alterations in neuroinflammation, axonal degeneration, myelin and macromolecular density, and iron were analyzed.

**Statistical Tests:**

Region‐based analysis (*p*‐value < 0.05, false discovery rate (FDR) corrected) and voxel‐based analysis (*p*‐value < 0.001 uncorrected, FDR‐corrected cluster extent = 5 voxels) were performed on 15 multisequence‐MRI metrics between the three groups.

**Results:**

The SoS atlas consisted of 35 regions and, after anatomical curation, the initial 506 tracts were refined to 78. Compared to HC, COVID‐P presented alterations in neuroinflammation‐related (mean: 41% of total alterations) and axonal degeneration‐related (31%) MRI metrics, while COVID‐R presented alterations of myelin‐related metrics (68%). COVID‐P alterations mainly affected the hindbrain (56%), while COVID‐R the hindbrain (39%).

**Data Conclusion:**

A novel tool, the SoS atlas, was developed to study the olfactory system and applied in combination with multisequence‐MRI metrics to investigate the mechanisms of COVID‐19 related anosmia.

**Evidence Level:**

3.

**Technical Efficacy:**

Stage 1.

## Plain Language Summary

The loss of smell, that is, anosmia, is a common symptom in COVID‐19, but the brain alterations behind it are still unclear. In this study, researchers developed the Sense of Smell (SoS) atlas, a new tool that included parts of the brain involved in olfaction. They applied the atlas to advanced MRI metrics of participants with persistent smell loss, participants who had recovered, and healthy controls. Results showed signs of neuroinflammation and axonal damage in persistent anosmia, and myelin changes in recovered anosmia. The SoS atlas provides a valuable tool to study smell‐related brain changes in COVID‐19 and other diseases.

## 1 Introduction

Olfactory dysfunction is the misperception of odors that can be both congenital and acquired, affecting individuals' daily functioning [1]. The variable loss of smell anosmia, can arise in neurological and neurodegenerative diseases, including dementia [2, 3]. During the COronaVIrus Disease 19 (COVID‐19) pandemic, anosmia emerged as an early symptom of acute SARS‐CoV‐2 infection and received a great deal of lay attention without a detailed scientific understanding of its underlying mechanisms [4, 5]. A comprehensive atlas of gray and white matter regions subtending olfaction was and is not available. This might be useful to assess tissue property differences associated with olfactory disfunction using multimodal‐MRI.

The olfactory circuit involves several brain regions [6]. The olfactory stimulus captured by receptor neurons and transmitted through the olfactory nerve is received by the olfactory bulb (OB) that projects to the primary olfactory cortex (POC), which includes the anterior olfactory nucleus (AON), the olfactory tubercle (OT), the piriform cortex (PirC), the entorhinal cortex (EC) and the amygdala (Amy) [7, 8]. From the POC, the information projects to the secondary olfactory cortex (SOC), which includes the thalamus (Thal), the hippocampus (Hippo), the hypothalamus (Hypo), the insula (Ins), and the orbitofrontal cortex (OFC). In addition to this well‐known pathway, the brainstem (BS) and the cerebellum (Cb), that is, the hindbrain (HB), have been shown to be involved in olfaction or to be affected (*postmortem*) in people who had experienced anosmia [9].

The HB is emerging as a hub to support brain functional dynamics and sensorimotor processing [10]. The BS serves as a gateway of many interconnected pathways, and hosts the autonomic centers involved in autonomic adjustments and visceral processes, regulated by olfactory stimuli [10, 11]. The Cb, known for its essential role in prediction, error detection and automatization of sensorimotor functions, is also involved in respiratory control and in odor detection and identification through the olfacto‐cerebellar pathway [12, 13, 14]. Different cerebellar regions, that is, the CrusI, LobVI, CrusII, LobXIII, and LobIX, have been reported to respond to unpleasant odors and odor processing [15, 16]. Moreover, the dentate nuclei (DN) were activated during a sniff‐and‐respond task, suggesting their role in olfaction [17].

Although a parcellation of the human POC using resting‐state functional MRI has been proposed, a structural atlas that includes the SOC, the HB and all connecting tracts remains unavailable [7].

During the COVID‐19 pandemic, MRI played a role for in vivo assessment of brain alterations in COVID‐19 subjects. Initial studies of subjects with anosmia focused on the OBs, for its relation to olfaction. Clinical cases in non‐hospitalized subjects showed hyperintensity and OBs atrophy using fluid attenuation inversion recovery (FLAIR) and T2‐weighted fat‐suppressed sequences; moreover, OB atrophy and alterations of the olfactory tract length and olfactory sulcus depth were reported [18, 19, 20]. These findings suggest that SARS‐CoV‐2 might invade the brain through the olfactory pathway, causing olfactory deficits that can persist months after the infection, known as persistent anosmia [21]. The use of clinical T1, T2, and FLAIR sequences demonstrated alterations in cortical gray matter regions (GM) involved in the olfactory circuit [22]. Diffusion tensor imaging (DTI) detected an increased mean diffusivity (MD) in the OFC [23].

The first aim of this work was to create an atlas of the olfactory circuit, the so‐called sense of smell (SoS) atlas, which includes GM regions of interest (ROIs) and white matter (WM) tracts, to facilitate localization of differences in areas involved in olfaction. Subsequently, the second aim was to investigate differences and localization of microstructural, inflammatory, myelin and iron imaging biomarkers in people with COVID‐19 persistent anosmia (COVID‐P), people who recovered from COVID‐19 anosmia (COVID‐R) and healthy control (HC), all vaccine naïve and scanned post‐acute phase.

## 2 Materials and Methods

### 2.1 Subjects and MRI Acquisition

Two different datasets were used: (1) the Human Connectome Project (HCP) dataset to create the atlas of axonal bundles involved in the olfactory circuit, taking advantage of its high spatial resolution; (2) a COVID‐19 MRI dataset to investigate brain changes in COVID‐P, COVID‐R, and HC.

#### 2.1.1 HCP Dataset

Ten HC (7 females, 22–35 years) were acquired with a Siemens Connectome Skyra 3 T scanner (https://www.humanconnectome.org). Diffusion weighted imaging (DWI) was acquired with a spin‐echo echo‐planar imaging (EPI) sequence with repetition‐time (TR) = 5520 ms, echo‐time (TE) = 89.5 ms, spatial resolution 1.25 × 1.25 × 1.25 mm^3^ and 288 volumes: 18 volumes with *b* = 0 s/mm^2^ and 270 volumes with *b* = 1000/2000/3000 s/mm^2^ (90 non‐collinear DWI directions/*b*‐value). Minimal pre‐processed DWI data was downloaded (EPI distortion, eddy current, and subject motion correction).

#### 2.1.2 COVID‐19 Dataset

Subjects were recruited in accordance with the declaration of Helsinki between June 2020 and March 2021 and gave their written informed consent as per local institution ethics approval. HC were included if they provided a self‐declaration of never having had COVID‐19 confirmed by an EDI IgM and IgG enzyme‐linked immunosorbent assay (ELISA) serum antibody negative test for SARS‐CoV‐2 at the time of scanning. COVID‐19 participants were included if they were beyond the acute phase of SARS‐COV‐2 infection, but during the acute phase had a positive Polymerase Chain Reaction (PCR) or lateral flow antigen test (LFT), had a clinically confirmed loss of sense of smell according to the University of Pennsylvania Smell Identification Test (UPSIT) test scoring < 33 and had not required hospitalization.

At the time of scanning, the UPSIT was repeated and participants were assigned to either of two groups: (1) COVID‐P, defined by UPSIT ≤ 25; (2) COVID‐R, defined by UPSIT ≥ 30.

All subjects had multisequence‐MRI, including 3D inversion recovery (IR), 3D fast field echo and spin‐echo EPI sequences and lasting 1 h15″ (Table 1), using a Philips Ingenia CX 3 T with a 32‐channel head coil. Exclusion criteria consisted of MR scanner contraindications (e.g., aneurysm clips, pacemakers, metallic implants, and pregnancy) and previous diagnosis of neurological conditions likely to affect imaging measures.

**TABLE 1:** MRI sequences of the COVID‐19 protocol.

Acquisition (time)	Voxel^a^ (mm)	FOV^a^ (mm)	# slices	Other info	TE/TR (ms)	FA
3DT1 (1′55″)	1 1 1	256 256 176	176 sag	3D‐FFE, TFE‐factor = 225, SENSE: no	3.1/6.9	8°
DWI (8′41″)	2 2 2	224 224 144	72 axial	SE, EPI‐factor = 55, SENSE = 2, *b*‐values = {0, 1000, 2000, 2800 s/mm^2^}, # directions = {4, 20, 20, 36}	96/6279	90°
IR (4′28″)	2 2 2	224 224 144	72 axial	SE, EPI‐factor = 55, SENSE = 2, [TI range]/dTI = [50–1910]/120 ms, 12 Tis	59/6885	90°
qMT (4′57″)	2 2 2	224 224 144	72 axial	SE, EPI‐factor = 55, SENSE = 2, offset = {96(*x*2), 13.7(*x*5), 3(*x*5)} MHz, FAs = {100(*x*2), 890(*x*5), 593(*x*5)}°	96/6287	90°
SPGR–multi TE (4′6″)	1 1 1	256 256 176	256 sag	3D‐FFE, SENSE: no, [TE range]/dTE = [2.3–25.4]/3.3 ms, 8 TEs	2.3–25.4/29	24°
B1‐DAM (30″/30″)	2 2 2	224 224 144	72 axial	SE, EPI‐factor = 55, SENSE = 2	59/15 × 10^3^	120°/60°

Abbreviations: B1‐DAM, B1 with double‐angle method; DWI, diffusion weighted imaging; EPI, echo planar imaging; FA, flip angle; FFE, fast field echo; FOV, field of view; IR, inversion recovery; qMT, quantitative magnetization transfer; sag, sagittal; SE, spin echo; SENSE, sensitive encoding; SPGR‐multi TE, spoiled gradient echo with multiple echo times; TE/TR, echo time/repetition time; TFE, turbo field echo; TI, inversion time.

^a^ Expressed as feet‐head (FH), anterior–posterior (AP), right–left (RL) directions for sagittal acquisition, RL‐AP‐FH for axial acquisitions.

### 2.2 SoS Atlas Creation

#### 2.2.1 GM‐ROI SoS Atlas

The atlas of the GM regions of the olfactory circuit (GM‐ROI SoS atlas) was created as follows. The OBs were manually segmented by three investigators (MG 6 years of expertise, EL 3 and EG 4). The OB ROIs were defined including voxels segmented by at least two investigators. Other regions were extracted from existing atlases: AON, OT and PirC from the *primary_olfactory_cortex_parcellation* [7], EC and Ins from the Juelich atlas, Amy, Hippo, and Thal from the FIRST atlas (http://www.fmrib.ox.ac.uk/fsl), Hypo from *hypothalamus_seg* (https://github.com/BBillot/hypothalamus_seg), OFC from the Brodmann atlas (https://afni.nimh.nih.gov/pub/dist/atlases/Brodmann_MM/), and BS from the Harvard‐Oxford‐Subcortical atlas (https://identifiers.org/neurovault.collection:262). Cerebellar ROIs were selected from the *spatially‐unbiased‐atlas‐template‐of‐the‐cerebellum‐and‐brainstem* (SUIT, https://github.com/diedrichsenlab/cerebellar_atlases): CrusI, CrusII, LobVI, LobVIII, LobIX, and DN. All regions were resampled to the MNI‐152 space and were divided into left (L) and right (R), except for the brainstem, for a total of 35 ROIs (Table 2).

**TABLE 2:** Regions of interest in the olfactory circuit.

	Labels (R‐L)	ROI	Abbreviation	From
Primary olfactory cortex	1–12	Olfactory bulb	OB	Manually segmented
	2–13	Anterior olfactory nucleus	AON	Primary olfactory cortex parcellation
	3–14	Olfactory tubercle	OT	Primary olfactory cortex parcellation
	4–15	Piriform cortex	PirC	Primary olfactory cortex parcellation
	5–16	Entorhinal cortex	EC	Juelich atlas
	6–17	Amygdala	Amy	FIRST atlas
Secondary olfactory cortex	7–18	Thalamus	Thal	FIRST atlas
	8–19	Hippocampus	Hippo	FIRST atlas
	9–20	Hypothalamus	Hypo	Hypothalamus seg
	10–21	Insula	Ins	Juelich atlas
	11–22	Orbitofrontal cortex	OFC	Brodmann atlas
Hindbrain	23	Brainstem	BS	Harvard‐Oxford Subcortical atlas
	24–30	Cerebellar crus I	Cb CrusI	SUIT atlas
	25–31	Cerebellar lobule VI	Cb LobVI	SUIT atlas
	26–32	Cerebellar crus II	Cb CrusII	SUIT atlas
	27–33	Cerebellar lobule VIII	Cb LobVIII	SUIT atlas
	28–34	Cerebellar lobule IX	Cb LobIX	SUIT atlas
	29–35	Dentate nucleus	DN	SUIT atlas

*Note*: Each row represents a different region, and for each region, the following information is indicated: the label number in the corresponding atlas (Labels (R‐L)), the name of the region (ROI) with its abbreviation (Abbreviation), and the atlas from which each region was extracted (From).

#### 2.2.2 WM‐Tract SoS Atlas

To create the atlas of the WM tracts of the olfactory circuit (WM‐Tract SoS atlas), the GM‐ROI SoS atlas was registered to each HCP subject's space applying a non‐linear transformation. For each subject the DWI data was used to perform a whole‐brain anatomically constrained tractography using probabilistic tractography and 30 million streamlines [24, 25]. From the whole‐brain tractogram, tracts connecting all combinations of pairs of the GM‐ROI SoS atlas regions were extracted and converted to the NIfTI format.

For each tract, only voxels with a value, that is, number of streamlines, higher than 5 were considered. Then, of the remaining tracts, only those common to all subjects were included in the next steps. To obtain anatomically realistic WM tracts and overcome some of the intrinsic limitations of tractography, the selected tracts were cleaned following two steps: (1) eliminate entire tracts recognized as false positives based on a priori anatomical assessment, that is, ipsilateral cerebro‐cerebellar tracts, intra‐hemispheric and inter‐hemispheric cerebellar tracts; (2) eliminate part of tracts, that is, spurious streamlines, whether considered anatomically invalid by using not‐ROIs, that is, regions where tract streamlines are not supposed to pass, defined as anatomical regions or self‐drawn planes [26]. To make the procedure generalizable across subjects, these regions were extracted from existing atlases in MNI‐152 space and registered into subject space, where each tract was visually checked to confirm it was correctly cleaned in all subjects. Two rules were used to constrain the cleaning process: (I) if three regions A, B, and C were connected, that is, from A to B and from B to C, only these tracts were considered while the longest tract connecting A to C was eliminated; (II) for homologous tracts, similar not‐ROIs were used, reflecting the brain symmetry.

After these steps, all tracts of each HCP subject were registered to the MNI‐152 space. To maintain the core of the tracts and reduce between‐subjects anatomical variability, a binary mask was created: each tract of each subject was binarized and averaged across subjects to generate a probabilistic mask with voxel values between 0 and 1, thresholded at 0.9. Then, for each tract, this binarized mask was applied to the corresponding tract weighted by the average number of streamlines across subjects. Finally, to create the atlas, each voxel had to be associated with only one tract: therefore, if a voxel belonged to multiple tracts, the one with the highest number of streamlines was chosen. A final refinement of the WM‐Tract SoS atlas was performed by removing very small tracts of less than 10 voxels (arbitrary lower limit).

### 2.3 Assessment of Anosmia Related Alterations in COVID‐19

#### 2.3.1 Multisequence‐MRI Processing

DWI, IR, quantitative magnetization transfer (qMT), and B1‐dual angle method (DAM) scans with identical EPI readout, underwent a joint pre‐processing pipeline involving noise, susceptibility distortion, and eddy‐current correction. Then, 15 multisequence‐MRI maps, reported with their biophysical meaning in Table 3, were extracted.

**TABLE 3:** Multimodal MRI metrics and their biophysical meaning.

	Variable	Acronym	Biophysical meaning
Axonal integrity	Fractional anisotropy from DTI model	FA	The degree of directional coherence that reflects microstructural integrity.
	Intra‐cellular volume fraction from NODDI model	*v*_intra_	Fraction of the neural tissue occupied by axons and dendrites.
	Probabilistic map of white matter form 3DT1	WM	Sensitive to white matter volume changes, including swelling or shrinkage due to inflammation or neurodegeneration.
Neuroinflammation	Mean diffusivity from DTI model	MD	The average across all spatial directions, sensitive to edema and inflammation, neurodegeneration.
	Isotropic volume fraction from NODDI model	*v*_iso_	Isotropic component of the diffusion‐weighted signal.
	Longitudinal relaxation time	qT1	Longitudinal relaxation sensitive to protons' environment, that is, myelin inflammation, cell density.
	Probabilistic map of gray matter form 3DT1	GM	Sensitive to gray matter volume changes, including swelling or shrinkage due to inflammation or neurodegeneration.
Myelin and macromolecular density	Diamagnetic susceptibility from *χ*‐separation model	*χ*_neg_	Spatial distribution of diamagnetic susceptibility (e.g., myelin) within brain tissues.
	*g*‐ratio from qMT and NODDI analysis	*g*‐ratio	Thickness of the myelin for a certain axon diameter.
	Short transverse relaxation time from qMT analysis	T2b	Transverse relaxation time sensitive to the autocorrelation time of bound protons.
	Macromolecular tissue volume from relaxometric map	MTV	Fraction of a voxel occupied by non‐water protons, associated with myelin and the presence of other macromolecules.
	Bound pool fraction from qMT analysis	BPF	Fraction of bound protons, for example, myelin or other macromolecules.
Iron	Effective transverse relaxation time from relaxometry	T2*	T2* of free protons, sensitive to local magnetic susceptibility, for example, iron concentration or iron forms (Fe^2+^ or Fe^3+^).
	Paramagnetic susceptibility from *χ*‐separation model	*χ*_pos_	Spatial distribution of paramagnetic susceptibility (e.g., iron) within brain tissues.
Iron and myelin	Quantitative susceptibility mapping from QSM reconstruction	QSM	Spatial distribution of magnetic susceptibility within brain tissues.

*Note*: Microstructure information can be obtained with diffusion tensor imaging (DTI) and neurite orientation dispersion and density Imaging (NODDI). Myelin‐related metrics can be obtained from quantitative magnetization transfer (qMT). Iron and myelin related metrics can be obtained from relaxometric maps, quantitative susceptibility mapping (QSM) reconstruction and *χ*‐separation model.

Specifically, from DWI, fractional anisotropy (FA) and MD were calculated using the DTI model, while the intra‐cellular volume fraction (*v*_intra_) and the isotropic volume fraction (*v*_iso_) using the neurite orientation dispersion and density imaging (NODDI) model [27, 28] (Dmipy‐https://github.com/AthenaEPI/dmipy).

From the IR scan, quantitative T1 (qT1) was extracted using a non‐linear least square fitting of the mono‐exponential IR signal model [29].

From B1‐DAM, B1 maps were calculated using actual flip angle images (MyRelax toolbox—https://github.com/fragrussu/MyRelax.git).

From qMT, magnetization transfer analysis was performed using qT1 and B1 maps and returning bound‐pool fraction (BPF) and the T2 of bound protons (T2b) [30, 31].

The *g*‐ratio was computed from the myelin volume fraction (MVF) and fiber volume fraction (FVF):

(1) $$g-\text{ratio}=\sqrt{1-\frac{\mathrm{MVF}}{\mathrm{FVF}}}$$

where

(2) MVF=kBPF, k=3.4

and

(3) $$\mathrm{FVF}=\mathrm{MVF}+\left(1-\mathrm{MVF}\right)\left(1-v_{\mathrm{iso}}\right)v_{\text{intra}}$$

The constant *k* was estimated by setting the mean *g*‐ratio to 0.7 in the forceps major of 5 randomly chosen HC (COVID‐19 dataset) [32].

A quantitative T2 map was calculated from the exponential fitting of the T2‐weighted signal from the IR data (short TE) and the non‐diffusion weighted T2 image from the DWI data (long TE) [33].

From the magnitude of data acquired with spoiled gradient echo sequences (SPGR) and B1 maps, transverse relaxation time of free protons (T2*) and macromolecular tissue volume (MTV) maps were extracted (MyRelax toolbox) [34].

Quantitative susceptibility mapping (QSM) was reconstructed from the complex SPGR data using MEDI (https://github.com/huawu02/MEDI_toolbox). *χ*_pos_ and *χ*_neg_ maps were computed with the χ‐separation method [35, 36].

### 2.4 Statistical Analyses

The multisequence‐MRI metrics were analyzed at the region and voxel‐level using the newly developed SoS atlas to investigate differences between groups. Furthermore, to highlight the complexity of COVID‐19 related anosmia, whole‐brain voxel‐level analysis was performed to investigate whether the alterations extend beyond the olfactory circuit.

#### 2.4.1 Region‐Based Analysis

The SoS atlas was registered to the subject space by inverting previously calculated transformations. For each subject, the robust mean of each one of the 13 metrics was calculated, per region and per tract, using the interquartile range rule and compared across groups using a linear model (LM) with age as a covariate (R software, http://www.rstudio.com/products/rstudio/download) (*p*‐value < 0.05, false discovery rate (FDR) corrected). The directionality of the alteration between the groups is reflected in the sign of the LM intercept.

#### 2.4.2 Voxel‐Based Analysis

##### 2.4.2.1 SoS Atlas

Voxel‐based morphometry (VBM) of GM and WM probability maps and voxel‐based analysis (VBA) of 13 multisequence‐MRI metrics were performed (SPM12, http://fil.ion.ucl.ac.uk/spm/).

VBM included these steps: GM and WM tissue segmentation in native space, DARTEL normalization, modulation, and smoothing with a Gaussian kernel of at least three times the voxel size [37, 38]. A general linear model (GLM), with intracranial volume and age as covariates, was performed to investigate structural differences between pairs of groups (*p*‐value < 0.001 uncorrected, FDR corrected using a cluster extent > 5 voxels).

VBA was performed for each of the 13 maps by normalizing them to the MNI‐152 space and smoothing them with a Gaussian kernel of at least three times the voxel size. GLM, with age as a covariate, was performed to investigate differences between pairs of groups (*p*‐value < 0.001 uncorrected, FDR corrected using a cluster extent > 5 voxels).

Then for each map, the extent of alterations was quantified by calculating the percentage of statistically significant voxels in each ROI and tract of the SoS atlas.

##### 2.4.2.2 Whole‐Brain

VBM and VBA were performed as explained in the previous section on the whole‐brain. The extent of alterations per area was quantified in each ROI of the Harvard‐Oxford cortical and subcortical structural atlases (https://identifiers.org/neurovault.collection:262), in each tract of the JHU‐atlas (http://www.fmrib.ox.ac.uk/fsl), and in each cerebellar ROI of the SUIT atlas.

##### 2.4.2.3 Localization and Biophysical Meaning

To obtain an overall spatial localization of brain changes, a probability map of the statistically significant alterations per region/voxel was calculated for each map. Specifically, the altered regions/clusters were binarized for each map and for each group comparison and averaged together.

Then, for each metric and for each level of analysis (i.e., region‐level and voxel‐level), the number of significantly different regions/voxels between groups was grouped by macro‐areas, namely POC, SOC, and HB for the analyses in the SoS atlas, and cortical, subcortical, and cerebellar regions plus forebrain and HB tracts for the whole‐brain analysis. These are represented with spider plots where radial lines are the metrics, concentric grid‐lines are the number of altered regions, and colored polygons the macro‐area involvement across metrics. Moreover, for each metric, the number of altered regions/voxels was represented as a bar plot, maintaining the direction of change. Finally, metrics were summarized by pie charts of localization or biophysical meaning, averaging between GM, WM, and the two levels of analysis.

## 3 Results

### 3.1 Participant Population

A total of 46 subjects were included in the study. One HC and one COVID‐R subject were discarded due to major imaging artifacts or missing sequences, leaving 44 subjects. Cohort details are reported in Table 4. Sex distribution was balanced across groups, whereas age was not.

**TABLE 4:** Demographic details for the COVID‐19 cohort.

	# of subjects	Sex (females/males)	Age (years)^a^	UPSIT^a^	Symptom to scan (days)^a^	COVID‐19 antibody (IgG/IgM) status
HC	17	8/9	39 ± 12 (25–69)	—	—	Negative
COVID‐R	19	14/5	38 ± 13 (25–56)	34 ± 2 (32–38)	127 ± 13 (108–146)	Positive
COVID‐P	8	7/1	52 ± 12 (33–65)	15 ± 5 (7–22)	190 ± 39 (123–246)	Positive
Significance (*p*‐value)	—	*p* = 0.11^b^	*p* = 0.03^c^	*p* < 0.001^d^	*p* < 0.001^e^	—

Abbreviations: COVID‐P, subjects with persistent anosmia; COVID‐R, subjects who recovered from anosmia; HC, healthy controls; IgG/IgM, immunoglobulin G/immunoglobulin M; UPSIT, University of Pennsylvania Smell Identification Test.

^a^ Measures are reported as mean ± standard deviation and range in brackets.

^b^ Fisher Exact Test was applied to compare the three groups.

^c^ Kruskal–Wallis test was applied to compare the three groups.

^d^ After testing for normality with the Shapiro–Wilk test, the Wilcoxon–Mann–Whitney test was applied to compare the two groups.

^e^ After testing for normality with the Shapiro–Wilk test, the *t*‐test was applied to compare the two groups.

### 3.2 SoS Atlas Creation

**FIGURE 1:**
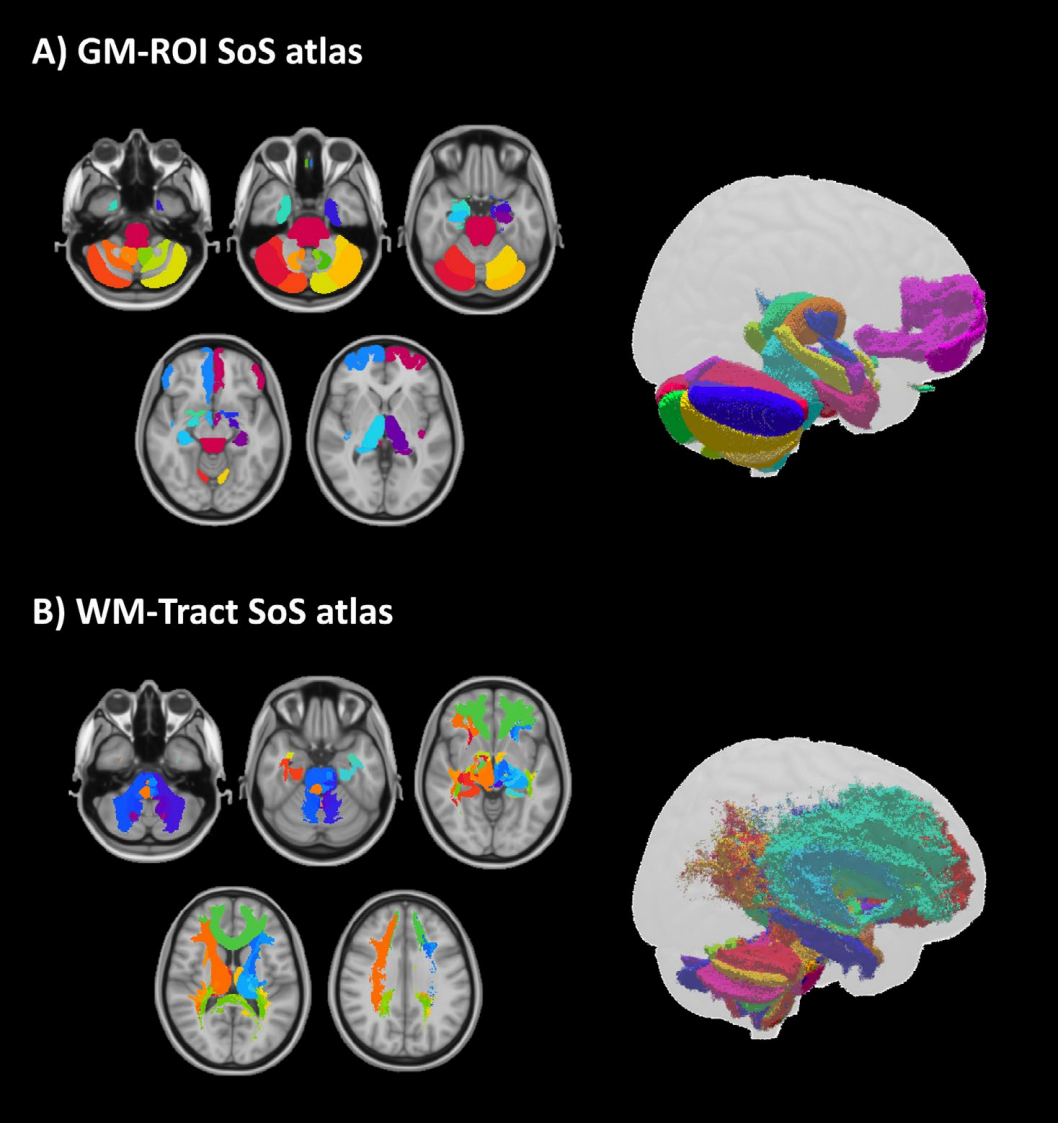
Sense of smell (SoS) atlas. The SoS atlas is visualized in the MNI‐152 space both selecting 5 axial slices (feet‐head direction) (left) and as a tridimensional representation (right). (A) The atlas of the gray matter regions of interest of the olfactory circuit (GM‐ROI SoS atlas). (B) The atlas of the white matter tracts (after curation) of the olfactory circuit (WM‐Tract SoS atlas).

The SoS atlas was successfully created, including GM regions and WM tracts (Figure 1), available on GitHub: https://github.com/marta‐gaviraghi/sense_of_smell_atlas. The GM‐ROI SoS atlas consists of 35 ROIs (Figure 1A).

**FIGURE 2:**
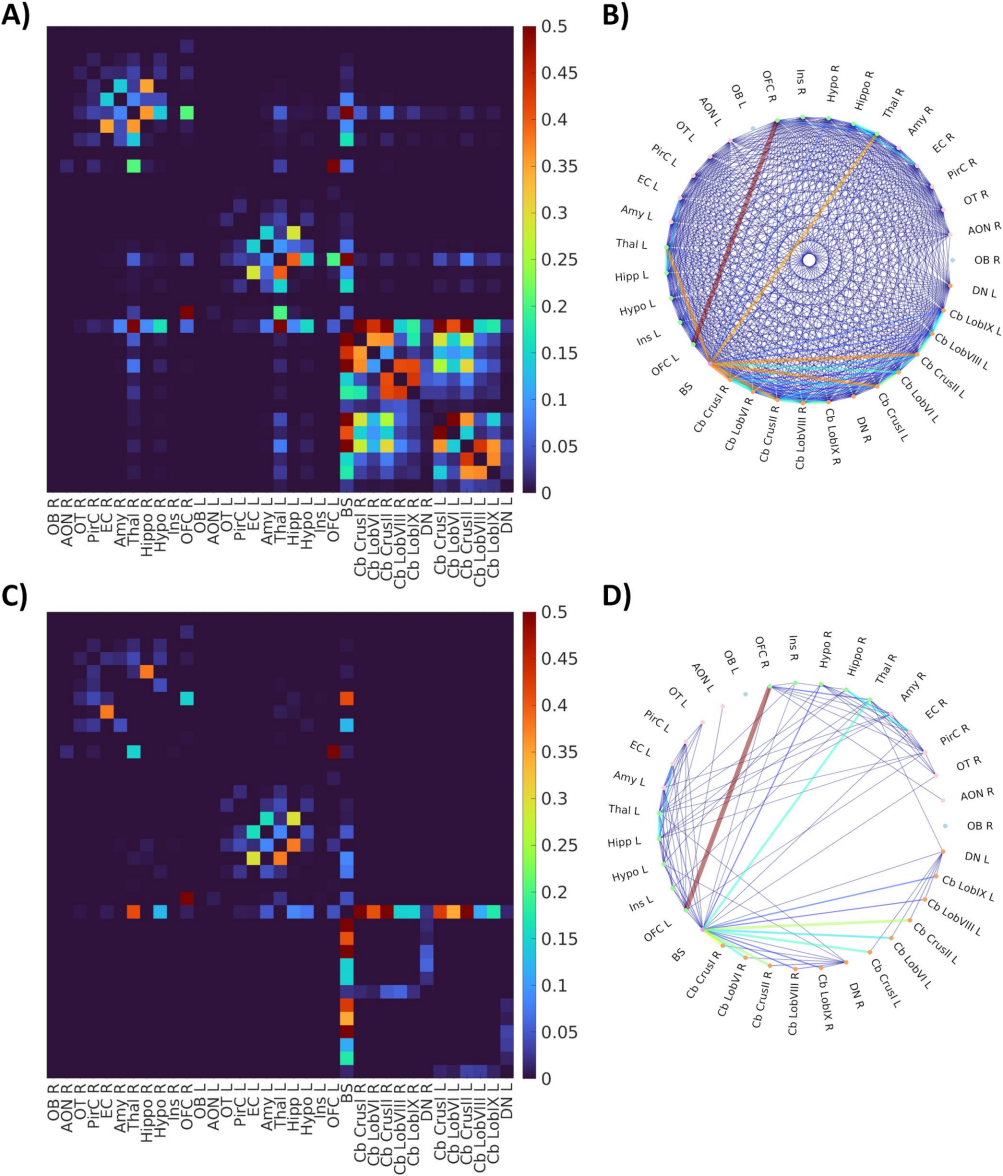
Structural connectivity and tract cleaning process. Structural connectivity before the curation (top row) and after the curation (bottom row). The left panel shows the structural connectivity matrix, where columns and rows are the gray matter regions, and values are the normalized number of streamlines connecting pairs of regions (A, C). The right panel shows connections between gray matter regions, with line thickness proportional to the strength of the connection (B, D).

The impact of the cleaning procedure is shown in Figure 2: of the original 506 tracts (Figure 2A,B), after step 1, 139 tracts survived, while after step 2, only 98 tracts remained (Figure 2C,D). Finally, the WM‐Tract SoS atlas includes 76 tracts (Figure 1B). Further details on the cleaning procedure are reported in Figure S1 and Table S1.

### 3.3 Anosmia Related Alterations in COVID‐19: Olfactory Circuit

Table 5 summarizes findings by presenting, for each metric and group comparison, only the most significant result (i.e., the lowest *p*‐value for the region‐based analysis or the highest absolute *T*‐score for the voxel‐based analysis). Details of all results for each group comparison are reported in Table S2 where results for the region‐based analysis are given in terms of the *p*‐value, the intercept, and the mean ± standard deviation for each metric and region, and in Table S3 where results for the voxel‐based analysis are given in terms of the *T*‐score and the mean ± standard deviation of each metric and affected region.

**TABLE 5:** Most significant regions for each metric in region‐based analysis and voxel‐based analysis.

		Region‐based analysis			Voxel‐based analysis		
		COVID‐P vs. HC	COVID‐P vs. COVID‐R	COVID‐R vs. HC	COVID‐P vs. HC	COVID‐P vs. COVID‐R	COVID‐R vs. HC
GM	Hypo R				0.39 ± 0.05 0.32 ± 0.04 *T* = 3.54		
	Cb LobVI L						0.48 ± 0.06 0.42 ± 0.06 *T* = 3.65
	Cb LobIX L					0.42 ± 0.04 0.34 ± 0.04 *T* = 3.72	
WM	EC L						0.011 ± 0.007 0.006 ± 0.002 *T* = 3.95
	Cb LobVI R				0.019 ± 0.004 0.014 ± 0.004 *T* = 3.90		
FA (a.u.)	AON R			0.17 ± 0.03 0.15 ± 0.02 *p* = 0.026 int. = 0.02			
	BS				0.37 ± 0.04 0.31 ± 0.04 *T* = 4.17	0.33 ± 0.05 0.30 ± 0.04 *T* = 3.87	
	Hypo L‐BS	0.37 ± 0.02 0.40 ± 0.02 *p* = 0.001 int. = −0.04	0.37 ± 0.02 0.39 ± 0.02 *p* = 0.002 int. = −0.04				
MD (×103 mm^2^/s)	OFC R		1.30 ± 0.14 1.11 ± 0.12 *p* = 0.004 int. = 0.20				
	OB L			1.90 ± 0.58 1.54 ± 0.38 *p* = 0.048 int. = 0.36			
	Cb LobIX R				1.76 ± 0.33 1.11 ± 0.27 *T* = 4.07	2.00 ± 0.33 1.29 ± 0.41 *T* = 3.87	
	Thal R‐BS	0.92 ± 0.06 0.86 ± 0.05 *p* = 0.002 int. = 0.09					
*v*_intra_ (a.u.)	Cb LobIX R				0.45 ± 0.06 0.33 ± 0.06 *T* = 4.07	0.43 ± 0.06 0.34 ± 0.06 *T* = 3.66	
	Thal R‐BS	0.40 ± 0.02 0.44 ± 0.03 *p* < 0.001 int. = −0.06					
	BS‐Cb CrusII R		0.50 ± 0.05 0.54 ± 0.03 *p* = 0.004 int. = −0.06				
*v*_iso_ (a.u.)	OB L			0.72 ± 0.22 0.53 ± 0.19 *p* = 0.012 int. = 0.19			
	Thal L		0.22 ± 0.09 0.12 ± 0.04 *p* = 0.005 int. = 0.10				
	Cb LobIX R				0.52 ± 0.12 0.24 ± 0.12 *T* = 4.12	0.58 ± 0.11 0.30 ± 0.18 *T* = 3.89	
	EC R‐Hippo R	0.15 ± 0.08 0.09 ± 0.03 *p* = 0.006 int. = 0.06					
qT1 (ms)	AON R			2.12 ± 0.79 2.28 ± 0.28 *p* = 0.007 int. = −0.33			
	OFC R		1.72 ± 0.18 1.60 ± 0.54 *p* = 0.002 int. = 0.27				
	Cb LobIX R				1.76 ± 0.20 1.45 ± 0.11 *T* = 3.75		
	Cb LobVI L						2.45 ± 0.38 2.07 ± 0.23 *T* = 3.80
	BS‐Crus II L	1.20 ± 0.09 1.13 ± 0.03 *p* = 0.005 int. = 0.07					
	Hippo R‐Hippo L					2.10 ± 0.28 1.67 ± 0.19 *T* = 4.43	
BPF (a.u.)	Hippo R						0.06 ± 0.01 0.05 ± 0.01 *T* = 3.95
	Hippo L		0.05 ± 0.01 0.06 ± 0.01 *p* = 0.001 int. = −0.01				
	Cb LobVI L					0.05 ± 0.01 0.04 ± 0.01 *T* = 3.66	
	Amy L‐Thal L			0.10 ± 0.02 0.10 ± 0.01 *p* = 0.001 int. = 0.01			
	Thal L‐Hypo L				0.08 ± 0.01 0.06 ± 0.01 *T* = 3.87		
*g*‐ratio (a.u.)	Hippo R						0.79 ± 0.03 0.74 ± 0.04 *T* = 4.03
	OB L	0.90 ± 0.12 0.76 ± 0.15 *p* = 0.018 int. = 0.21					
	EC L					0.84 ± 0.04 0.79 ± 0.04 *T* = 3.71	
	Hypo R‐BS			0.76 ± 0.05 0.77 ± 0.02 *p* = 0.002 int. = −0.03			
	Thal L‐Hypo L				0.78 ± 0.04 0.72 ± 0.04 *T* = 3.36		
	EC L‐Hippo L		0.73 ± 0.03 0.72 ± 0.06 *p* = 0.003 int. = 0.04				
T2b (ms)	Cb LobVI L			9.44 ± 0.44 9.97 ± 0.38 *p* < 0.001 int. = −0.52			15.42 ± 2.85 12.13 ± 1.99 *T* = 3.99
	Ins R‐Thal L					13.62 ± 2.33 11.00 ± 1.13 *T* = 3.86	

	OT R‐PirC R		11.80 ± 4.99 9.96 ± 3.01 *p* = 0.022 int. = 3.62				
Thal R‐BS	10.26 ± 0.30 10.58 ± 0.37 *p* = 0.009 int. = −0.41					
T2*(ms)	PirC R‐Thal R	38.11 ± 4.80 45.19 ± 2.64 *p* = 0.001 int. = −6.00					
	PirC R‐Hippo R			44.36 ± 2.75 47.84 ± 3.69 *p* = 0.003 int. = −3.47			
	Thal R‐Hippo L		37.95 ± 5.94 40.89 ± 3.78 *p* = 0.033 int. = −4.96				
MTV (a.u.)	OT R		0.19 ± 0.03 0.15 ± 0.06 *p* = 0.042 int. = 0.06				
*χ*_pos_ (ppm)	Thal R		0.01 ± 0.01 0.01 ± 0.01 *p* = 0.022 int. = −0.01				
	PirC R‐Hypo R					0.06 ± 0.03 0.04 ± 0.02 *T* = 3.92	
	Amy R‐Amy L			0.02 ± 0.01 0.03 ± 0.01 *p* = 0.008 int. = −0.01			
	Hypo L‐BS						0.04 ± 0.02 0.03 ± 0.01 *T* = 3.64
*χ*_neg_ (ppm)	AON R						0.04 ± 0.02 0.03 ± 0.01 *T* = 3.39
	PirC R‐OFC R		0.02 ± 0.01 0.03 ± 0.01 *p* = 0.009 int. = −0.01	0.03 ± 0.01 0.02 ± 0.01 *p* = 0.042 int. = 0.004			
ǪSM (ppm)	Amy L				−0.03 ± 0.01 −0.05 ± 0.02 *T* = 3.43		
	EC R‐BS					0.001 ± 0.014 −0.02 ± 0.01 *T* = 3.82	
	OFC R‐OFC L	0.001 ± 0.004 −0.001 ± 0.003 *p* = 0.031 int. = 0.004					
	AON R‐OFC R			−0.02 ± 0.01 −0.01 ± 0.01 *p* = 0.028 int. = −0.01			
	Thal R‐Hippo L		−0.01 ± 0.01 −0.02 ± 0.01 *p* = 0.006 int. = 0.01				
	EC L‐Hippo L						−0.03 ± 0.02 −0.05 ± 0.02 *T* = 3.85

*Note*: The most significant regions are reported for both the region‐based analysis and voxel‐based analysis. For each group comparison (COVID‐P vs. HC, COVID‐P vs. COVID‐R, and COVID‐R vs. HC) and for each metric, the region corresponding to the lowest *p*‐value is reported for the region‐based analysis, and the region with the highest *t*‐score is reported for the voxel‐based analysis. Each cell shows, for the region, the metric's mean ± standard deviation for the first and second group, the *p*‐value and the intercept for the region‐based analysis and *t*‐score for the voxel‐based analysis.

**FIGURE 3:**
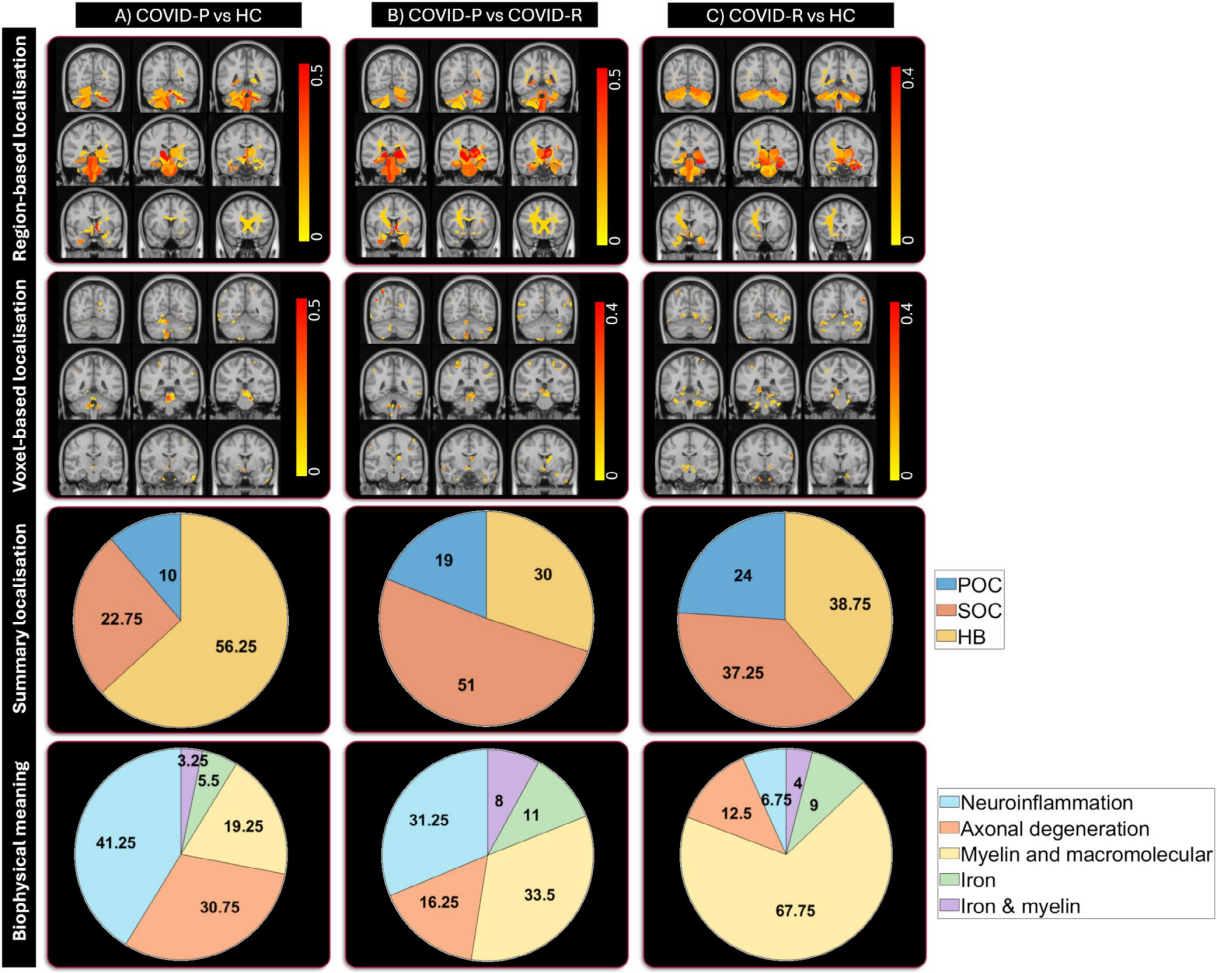
Spatial localization and biophysical meaning of brain alterations. The first two rows show the brain alterations overlaid on coronal T1 images in the MNI‐152 space (selection of sequential posterior to anterior sections): Region‐based analysis (first row) and voxel‐based analysis (second row). The last two rows show pie charts summarizing the localization and biophysical meaning of the alterations, averaged between gray and white matter and the two levels of analysis (i.e., region‐based and voxel‐based). Localization shows the alterations divided into primary olfactory cortex (POC), secondary olfactory cortex (SOC), and hindbrain (HB) (third row), while the alterations' distribution is summarized into biophysical meanings across MRImultimodal maps (fourth row). All results are reported between (A) COVID‐P and HC, (B) COVID‐P and COVID‐R, (C) COVID‐R and HC. COVID‐P, people with COVID‐19 related persistent anosmia; COVID‐R, people who recovered from COVID‐19 related anosmia; HC, healthy controls.

Figure 3 shows for each group comparison the spatial localization and the amount of altered metrics in MNI space, while the pie charts illustrate the corresponding macro‐area distribution and biophysical meaning of alterations (see also Tables S4–S6). Compared to HC, COVID‐P showed mainly neuroinflammation (41%) and axonal degeneration (31%), predominantly affecting the HB (56%); while COVID‐R had myelin‐related (68%) alterations, mostly involving HB (39%).

**FIGURE 4:**
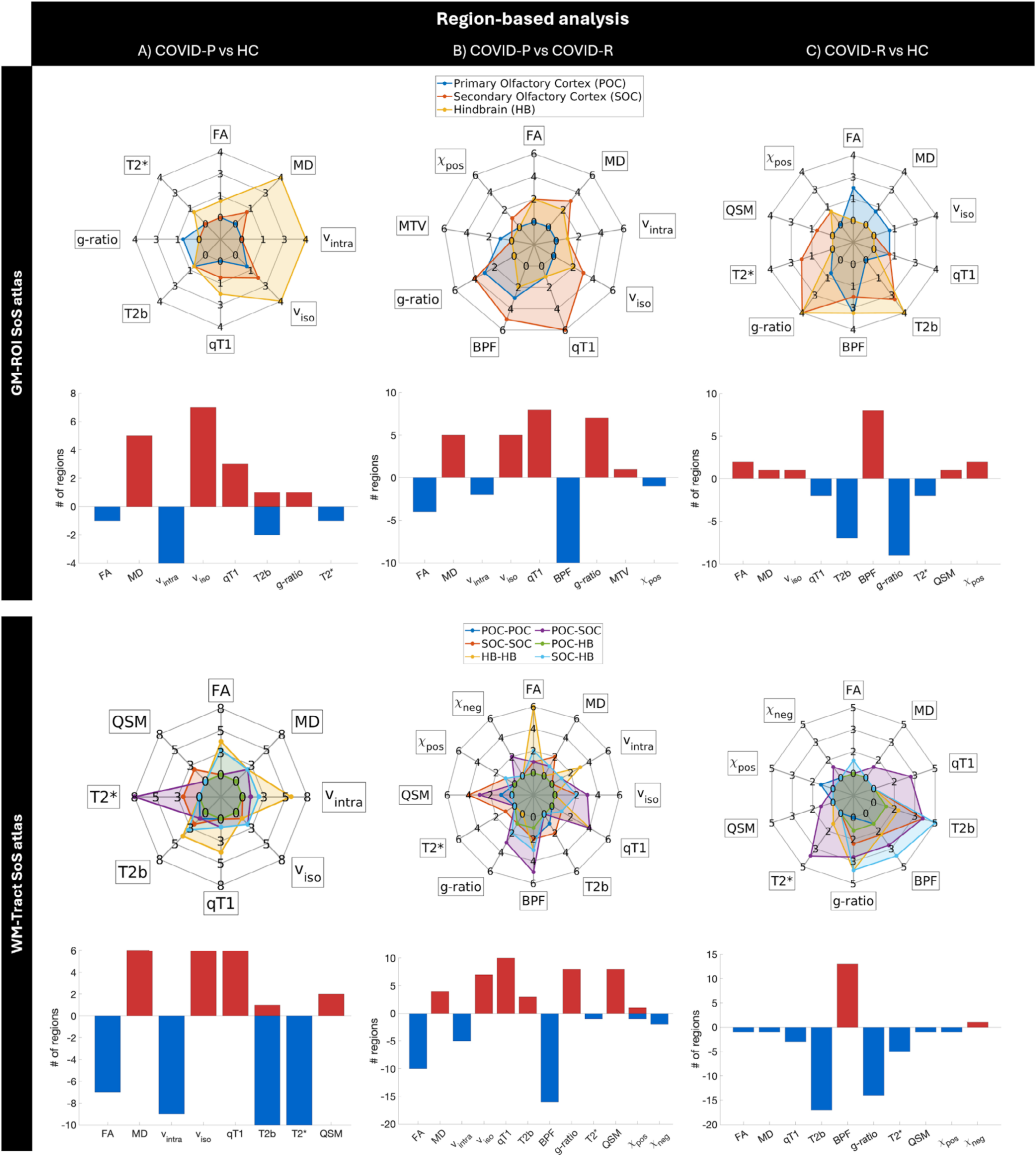
Region‐based analysis in the SoS atlas. Spider charts and bar plots show the number of regions with significant differences between each pair of groups: (A) COVID‐P versus HC, (B) COVID‐P versus COVID‐R, and (C) COVID‐R versus HC. The first two rows illustrate the region‐based analysis in the GM‐ROI SoS atlas, while the last two rows in the WM‐Tract SoS atlas, where regions are grouped by primary olfactory cortex (POC)—blue areas, secondary olfactory cortex (SOC)—red areas, and hindbrain (HB)—yellow areas. In the spider charts, radial axes represent multisequence‐MRI metrics. For example, in COVID‐P versus HC, the HB area (yellow) extends more, indicating widespread alterations across different metrics (multiple radii). In the bar plots, blue bars represent a decrease, while red bars represent an increase of the number of MRI metrics altered in the first group compared to the second. BPF, bound‐pool fraction; COVID‐P, people with COVID‐19 related persistent anosmia; COVID‐R, people who recovered from COVID‐19 related anosmia; FA, fractional anisotropy (FA); *g*‐ratio, T2*, MTV, macromolecular tissue volume; HC, healthy controls; MD, mean diffusivity; QSM, quantitative susceptibility mapping; qT1, quantitative T1; T2b, quantitative T2 of the bound pool component; *v*_intra_, intra‐cellular volume fraction; *v*_iso_, isotropic volume fraction; *χ*_neg_, negative susceptibility; *χ*_pos_, positive susceptibility.

Figure 4 shows region‐based results, with spider charts and bar plots quantifying the number of altered regions/tracts of the SoS atlas (see also Figures S2–S5).

COVID‐P compared to HC showed lower FA, *v*_intra_, T2*, and greater MD, *v*_iso_, qT1, *g*‐ratio, QSM, mostly located in HB regions and tracts connecting POC with SOC and intra HB (Table 5). T2b was higher in the left OB but lower in the right OFC, right DN, and multiple tracts.

COVID‐P compared to COVID‐R showed lower FA, *v*_intra_, BPF, T2*, and *χ*_neg_ while higher MD, *v*_iso_, qT1, T2b, *g*‐ratio, QSM, and MTV, mostly located in SOC regions and tracts connecting POC with SOC and intra HB tracts. *χ*_pos_ was lower in the right Thal and in the right OFC‐BS tract and was greater and in the left Hippo‐left Ins tract.

COVID‐R compared to HC showed lower qT1, T2b, *g*‐ratio, and T2* while higher *v*_iso_, BPF, and *χ*_neg_, mostly located in SOC and HB regions and the tracts connecting POC with SOC, SOC with HB, and intra HB tracts. FA was higher in the right AON and right Amy, but lower in the left Hypo‐BS. MD was greater in the left OB, but lower in the left PirC‐left OFC tract. QSM was higher in the right Hypo, but lower in the right AON‐right OFC. *χ*_pos_ was higher in the left OFC and left Cb CrusII but decreased in the connection between contralateral Amy.

**FIGURE 5:**
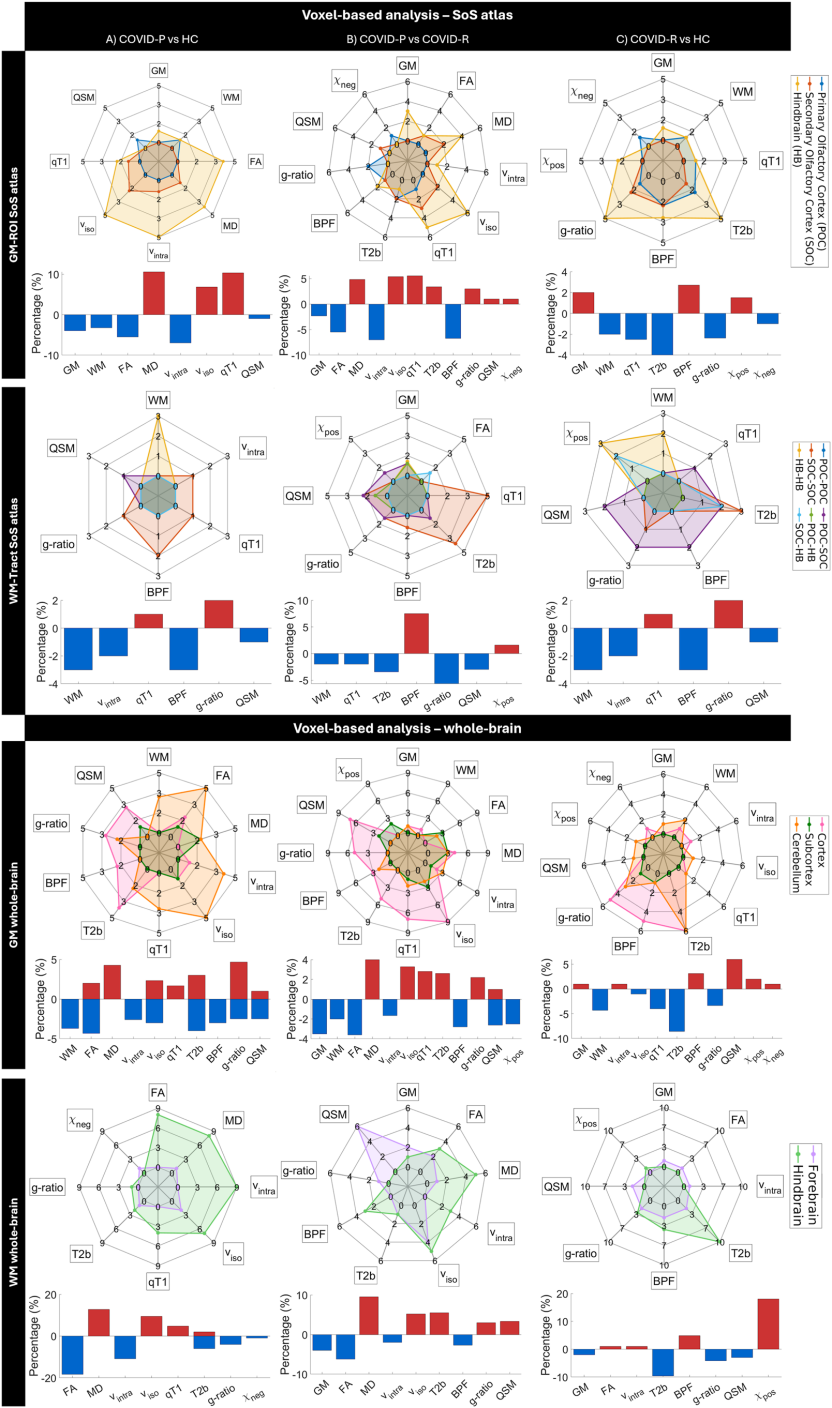
Voxel‐based analysis in the SoS atlas and in the whole‐brain. Each spider chart shows the number of regions with significant differences between each pair of groups, while each bar plot shows the mean percentage of altered voxels in significantly affected regions between each pair of groups: (A) COVID‐P versus HC, (B) COVID‐P versus COVID‐R, and (C) COVID‐R versus HC. The rows illustrate the voxel‐based analysis respectively in the GM‐ROI SoS atlas and in the WM‐Tract SoS atlas, where regions are grouped by primary olfactory cortex (POC), secondary olfactory cortex (SOC), and hindbrain (HB), in the GM, where regions are grouped by cortex, subcortex and cerebellum represented in different colors, and the WM of forebrain and hindbrain. In the spider charts, radial axes represent multisequence‐MRI metrics. In the bar plots, blue bars represent a decrease, while red bars represent an increase of the number of MRI metrics altered in the first group compared to the second. BPF, bound‐pool fraction; COVID‐P, people with COVID‐19 related persistent anosmia; COVID‐R, people who recovered from COVID‐19 related anosmia; FA, fractional anisotropy (FA); *g*‐ratio, T2*, MTV, macromolecular tissue volume; HC, healthy controls; MD, mean diffusivity; QSM, quantitative susceptibility mapping; qT1, quantitative T1; T2b, quantitative T2 of the bound pool component; *v*_intra_, intra‐cellular volume fraction; *v*_iso_, isotropic volume fraction; *χ*_neg_, negative susceptibility; *χ*_pos_, positive susceptibility.

Figure 5 shows the voxel‐based results, with spider charts and bar plots quantifying the mean percentage of altered voxels in regions/tracts of the SoS atlas (see also Figure S6).

COVID‐P compared to HC showed lower GM and WM, FA, *v*_intra_, BPF, and QSM, while higher MD, *v*_iso_, qT1, and *g*‐ratio, mostly located in the HB regions and intra HB and intra SOC tracts. Notably, the Cb LobIX R exhibited the greatest reduction (25%) in the *v*_intra_. The WM‐Tracts involving BS and Cb showed the most significant differences in WM.

COVID‐P compared to COVID‐R showed lower GM, FA, *v*_intra_, BPF, and *χ*_pos_, while higher MD, *v*_iso_, qT1, T2b, *g*‐ratio, and *χ*_neg_, mostly located in the HB regions and intra SOC tracts. T2b was higher in the left OB but lower in the right OFC, right DN, and multiple tracts. QSM was generally lower in the tracts, except for right Thal‐OFC and right Ins‐OFC tracts and for the right Thal. The mostly affected GM‐ROIs were the left Amy, right Hypo, and right Cb LobIX. Notably, the right Hypo exhibited the greatest change (30%) in qT1. The mostly affected WM‐Tracts was the right PirC‐Hypo in *χ*_pos_ (10%).

COVID‐R compared to HC showed lower WM, qT1, T2b, *g*‐ratio, QSM, and *χ*_neg_, while higher GM, BPF, and *χ*_pos_, mostly located in HB regions and the tracts connecting POC with SOC, and intra HB. The mostly affected GM‐ROI was the right Cb LobVI. The mostly affected WM‐Tracts were the right PirC‐right OFC (10%) for *g*‐ratio and right Ins‐BS (13%) for T2b.

### 3.4 Anosmia Related Alterations in COVID‐19: Whole‐Brain

Figure 5 illustrates the voxel‐based results at the whole‐brain level. The metric alterations observed at the whole‐brain level are consistent with those observed in the olfactory circuit (Figure S7 and Results in Supporting Information).

## 4 Discussion

This work presents a comprehensive atlas of the olfactory circuit, with regions and tracts designed to be anatomically curated. For what concerns WM tracts connecting GM regions, each tract was carefully curated to be anatomically realistic, following a robust and generalizable procedure.

The newly developed SoS atlas was used in combination with multisequence‐MRI metrics to localize and characterize brain alterations in COVID‐19 anosmia, as a first clinical application. Results showed that neuroinflammation and myelin alterations involve not only the OBs but also a large‐scale network involving multiple forebrain and HB regions. Results showed that not only POC and SOC are affected, but also that the HB has large alterations and therefore must play a crucial role in processes occurring in COVID‐P and COVID‐R.

### 4.1 The Olfactory Circuit Atlas: Methodological Considerations

Tractography was used to reconstruct the WM‐Tract SoS atlas, as it is the only technique applicable in humans in vivo, despite its well‐known intrinsic limitations (e.g., high number of false positives). These were overcome at best by curation—eliminating or modifying tracts based on anatomical knowledge to ensure anatomical reliability. It is noteworthy that starting from 506 tracts, 98 were selected and collapsed into 76 tracts of the WM‐Tract SoS atlas. This meticulous, one‐tract‐at‐a‐time curation process provides an anatomically realistic atlas, which provides the best outcome considering that there is no method for in vivo *in‐humans* non‐invasive validation. The SoS atlas could enable a more specific characterization of olfactory circuit changes in neurological and neurodegenerative diseases, like Alzheimer's, Parkinson's, and Huntington's diseases [2, 3].

### 4.2 Axonal Degeneration and Neuroinflammation in COVID‐19

People who experienced COVID‐19 related anosmia showed differences at the region‐ and voxel‐level, possibly indicating structural alterations, changes related to inflammation, microstructure, myelin, and iron content, which point to several potential underlying pathological mechanisms affecting the olfactory circuit. Timings of such changes cannot be inferred by the present cross‐sectional study.

COVID‐P compared to HC showed higher MD, *v*_iso_, and qT1, along with lower FA, WM volume, and *v*_intra_, which may respectively indicate the presence of inflammation and axonal degeneration. This is further supported by lower T2*, explainable by iron accumulation or changes in tissue magnetic properties, and a generally lower T2b, related to macromolecular density/structure, including myelin [39, 40]. QSM alterations, linked to altered iron homeostasis (Fe^3+^ but not Fe^2+^), appear inconsistent, suggesting the presence of neuroinflammation rather than altered iron homeostasis per se. Brain regions and tracts mostly affected by inflammation and axonal changes are directly connected to the HB. Consistent with the literature, this could be due to preferential viral invasion of the HB by SARS‐CoV‐2, as reported in post‐mortem studies [9, 41]. Alternatively, it could reflect infectious or para‐infectious mechanisms, such as autoimmune responses. Indeed, Paterson et al. [42] reported a delayed effect, a lack of viral positivity in cerebrospinal fluid and brain biopsies, and other imaging changes that show a characteristic pattern, for example, HSV‐1 encephalitis.

COVID‐P compared to COVID‐R showed, mostly in SOC and HB, lower FA and *v*_intra_ suggesting potential axonal damage in COVID‐P. In COVID‐P, higher MD, *v*_iso_, and qT1 support the presence of neuroinflammation, while a lower BPF and longer T2b suggest either myelin loss, supported by increased QSM, or a change in myelin microstructure, supported by increased *g*‐ratio. These findings, despite the limited sample size, are in line with literature reports of neuroinflammation in COVID‐P, while adding new evidence of potential myelin‐related alterations and axonal degeneration persisting after the SARS‐CoV‐2 acute infection [43]. While assessments of myelin changes are still an open research question in human studies, they have been confirmed in animal models comparing influenza and SARS‐CoV‐2 [44, 45].

### 4.3 Neuropathological Implications of COVID‐19 Related Alterations

Reassuringly, COVID‐R presented higher BPF and lower *g*‐ratio and T2b, mostly in SOC and HB, hence indicating a higher or altered macromolecular density/microstructure (e.g., myelin content/thickness). These results suggest that people who recover from COVID‐19 acute symptoms of anosmia may either have brain tissue characteristics that make them less predisposed to persistent symptoms or exhibit compensatory processes that support their functional recovery [46]. Even if we did not detect changes in inflammation metrics in COVID‐R at the time of scanning, inflammation might have been present during the anosmia phase to trigger a remyelination process while also resolving itself. Indeed, inflammation plays a crucial role in creating a favorable environment for myelin repair [46]. Longitudinal studies with a comprehensive multisequence‐MRI protocol should be performed for a correct interpretation of the observed changes, including whether myelin and axonal damage are indeed reversible.

### 4.4 The Predominance of Hindbrain Alterations

The predominance of HB alterations over POC and SOC observed in COVID‐P raises an interesting question on the role—or susceptibility—of the HB to neuroinflammation and neurodegeneration processes.

Previous findings have demonstrated BS involvement in COVID‐19, recognizing that the BS is a preferential viral route to the central nervous system and may be responsible for respiratory illness [47]. Interestingly, our data confirm BS alterations also in COVID‐19 persistent anosmia.

We also found COVID‐19 related alterations in cerebellar regions extending beyond those included in the SoS atlas, underlying the importance of considering the whole‐brain, as well as the SoS atlas for complementary information, rather than limiting investigations only to the forebrain. While not often considered as part of the olfactory circuit, the cerebellum influences odor detection, sniffing behavior, and chemosensory processing [14, 48]. The impact of the viral infection on the cerebellum can be explained by considering the mechanisms of SARS‐CoV‐2 for cellular entry. The SARS‐CoV‐2 spike protein binds to the angiotensin‐converting enzyme 2 receptor to facilitate cellular entry, assisted by cysteine proteases; considering the higher expression of the cathepsin protease gene in the cerebellum, this may explain its high susceptibility to SARS‐CoV‐2 infection and the predominant alterations that we detected in the cerebellum within and beyond the SoS atlas [49, 50].

### 4.5 Limitations

Future studies should increase the sample size and account for age‐related variability to further validate the WM‐Tract SoS atlas. Moreover, a potential limitation of this study is the small sample size of the COVID‐19 dataset, especially considering the number of metrics and ROIs analyzed. This could reduce statistical power; however, all analyses were corrected for multiple comparisons using FDR. Larger multi‐center and longitudinal studies are needed to confirm and generalize our findings.

## 5 Conclusions

The SoS atlas was developed and is available for assessing olfactory alterations in neurological/neurodegenerative disorders. Its first clinical application in COVID‐19 related anosmia showed the prominent involvement of the hindbrain, supporting its crucial role in explaining persistent anosmia after SARS‐CoV‐2 infection. We suggest, in line with literature, that COVID‐P subjects have ongoing neuroinflammation and axonal degeneration, whereas myelin changes in COVID‐R may suggest compensatory mechanisms that should be further investigated with longitudinal studies. Tackling inflammation and myelin repair may be an important avenue for future clinical trials, and the SoS atlas with a multisequence‐MRI protocol to assess inflammation, neurodegeneration, and myelin changes could provide invaluable outcome measures.

## Conflicts of Interest

M.S.Z. has received honoraria for a lecture each in the last 3 years from UCB Pharma, GSK, Cygnet healthcare. JM has recieved institutional funding from NovoNordisk and Rhythm Pharmaceuticals and research funding form the NIHR and the Society for Endocrinology.

## Supporting information


**Data S1:** jmri70128‐sup‐0001‐supinfo.zip.

## Data Availability

The SoS atlas is available online at the GitHub link: https://github.com/marta‐gaviraghi/sense_of_smell_atlas. The data that supports the findings of this study will be openly available on the EBRAINS platform.
