# Supplementary material for: The Sense of Smell (SoS) Atlas: Its Creation and First Application to Investigate COVID‐19 Related Anosmia With a Comprehensive Quantitative MRI Protocol
Source: J Magn Reson Imaging. 2025 Oct 3;63(2):574–93. doi: 10.1002/jmri.70128 (PMC12811004; doi:10.1002/jmri.70128)
Supplement: Supplementary file 1 — Data S1: jmri70128‐sup‐0001‐supinfo.zip. [file JMRI-63-574-s001.zip › Supplementary_figure.docx]

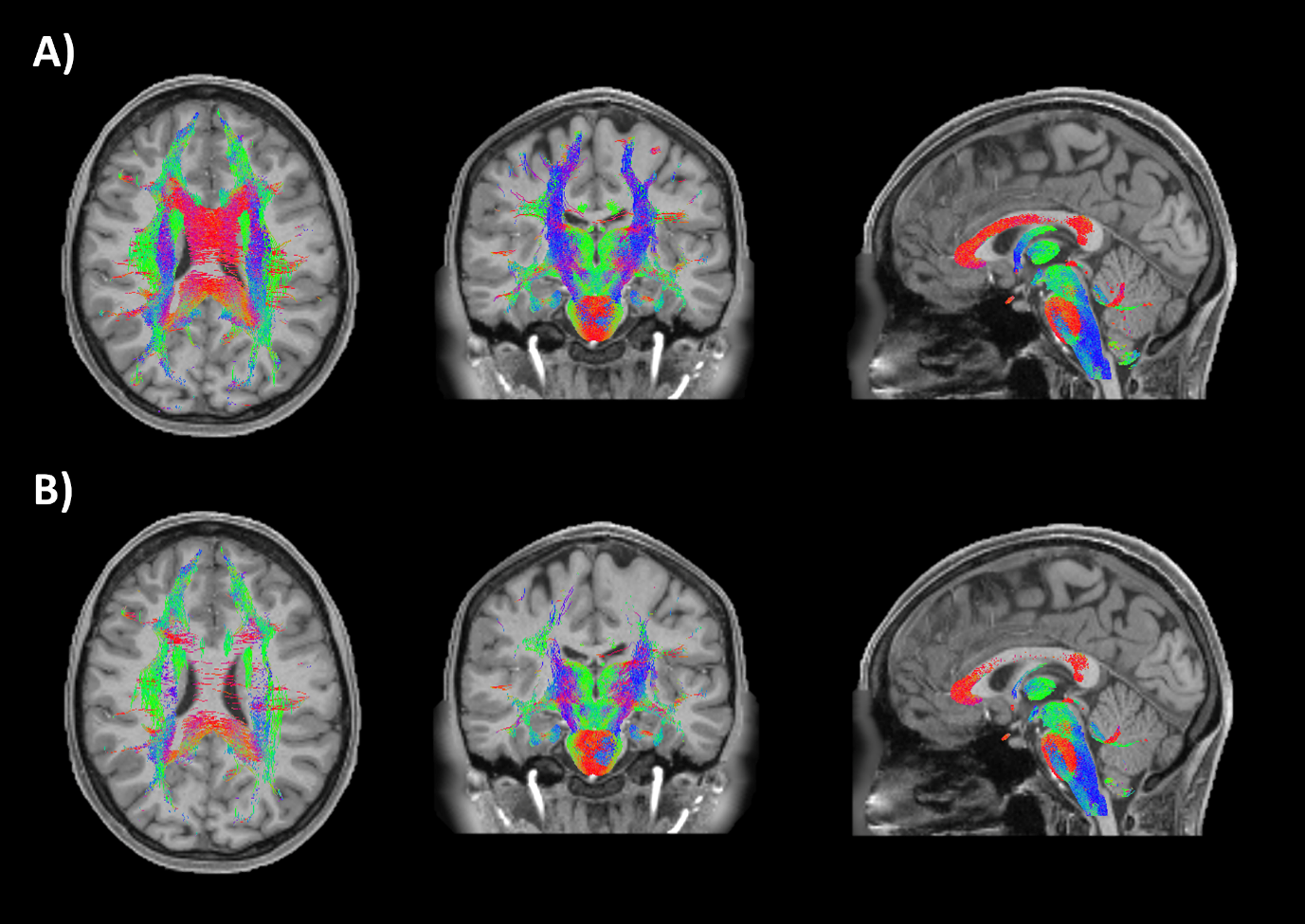


**Supplementary Fig.1:** **Tractogram of the olfactory circuit.** The tractogram of the olfactory circuit before **A**) and after **B**) the cleaning procedure in a randomly chosen subject. Images are in the native space using the radiological view. Notably the curation process effectively eliminated most of the false positives crossing midline through the corpus callosum and the fornix.


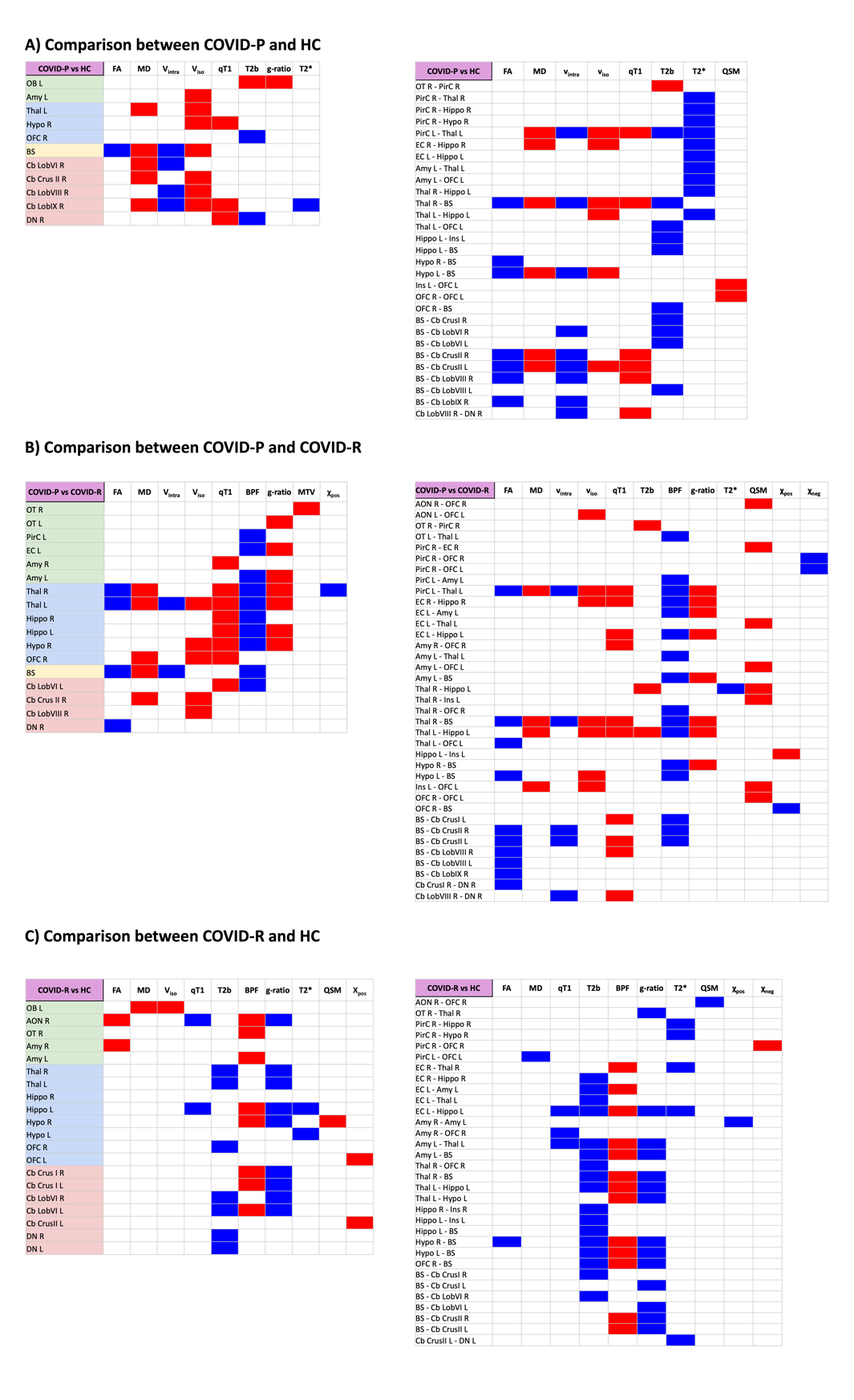


**Supplementary Fig.2:** **Region-based analysis between groups using the SoS atlas.** The table on the left reports the grey matter regions (GM-ROI) SoS atlas while the table on the right reports the white matter tracts (WM-Tract) SoS atlas showing significant differences (linear model, p<0.05). For each comparison, **A**) COVID-P vs HC, **B)** COVID-P vs COVID-R, **C)** COVID-R vs HC, blue cells represent a significant decrease while red cells represent an increase of the MRI maps in the first group. The analysed MRI maps are: fractional anisotropy (FA), mean diffusivity (MD), intra-cellular volume fraction (v_intra_), isotropic volume fraction (v_iso_), quantitative T1 (qT1), quantitative T2 of the bound pool component (T2b), bound-pool fraction (BPF), g-ratio, T2*, macromolecular tissue volume (MTV), quantitative susceptibility mapping (QSM), positive susceptibility (χ_pos_) and negative susceptibility (χ_neg_).

HC: healthy controls, COVID-P: people with COVID-19 related persistent anosmia, COVID-R: people who recovered from COVID-19 related anosmia.


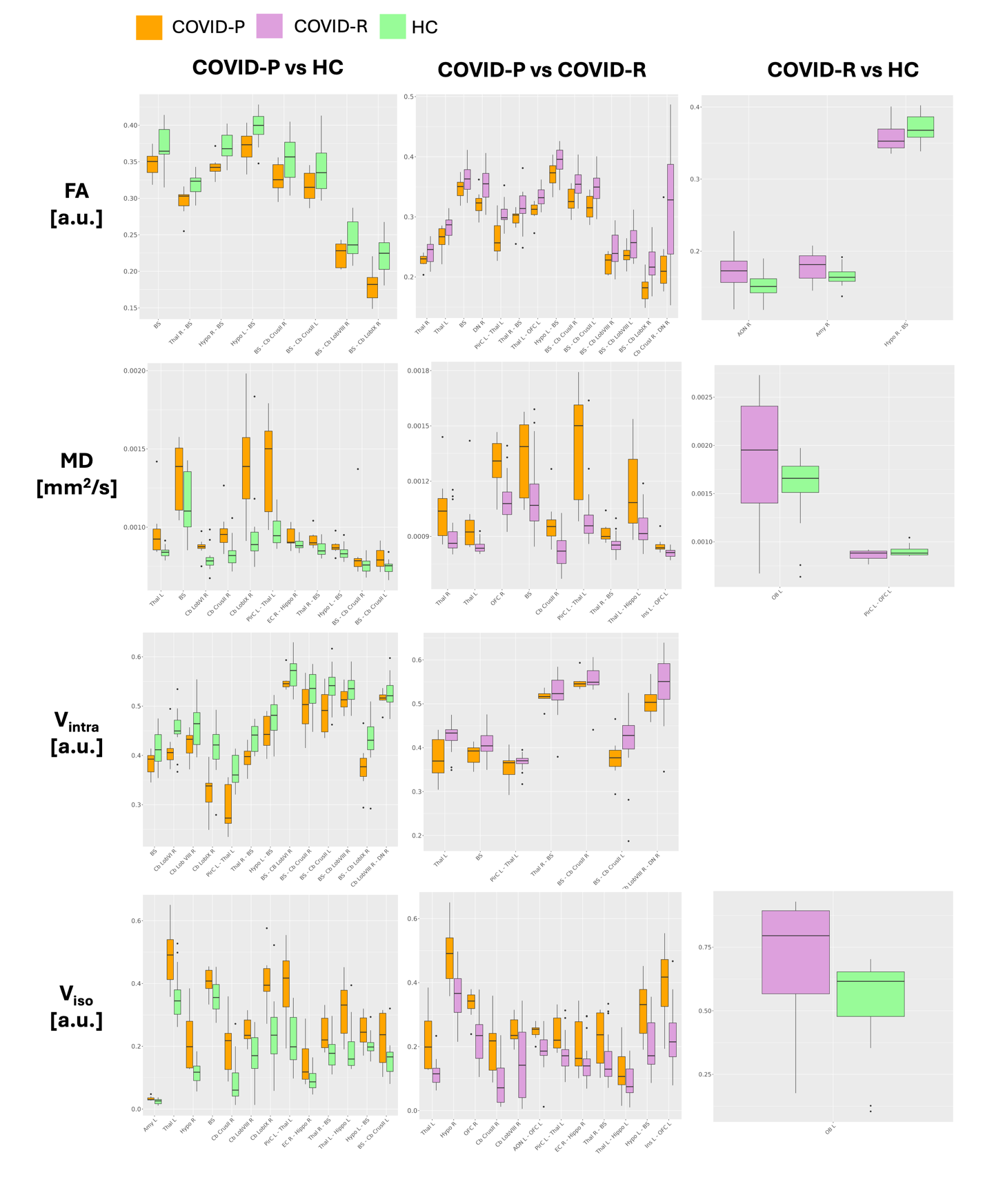


**Supplementary Fig.3:** **Boxplots of microstructural metrics.** Boxplots of the microstructural MRI metrics in the regions and tracts of the SoS atlas in HC (green), COVID-P (orange) and COVID-R (pink). Only significant differences (p<0.05) are reported for each comparison. The metrics are fractional anisotropy (FA), mean diffusivity (MD), intra-cellular volume fraction (v_intra_) and isotropic volume fraction (v_iso_).

HC: healthy controls, COVID-P: people with COVID-19 related persistent anosmia, COVID-R: people who recovered from COVID-19 related anosmia.


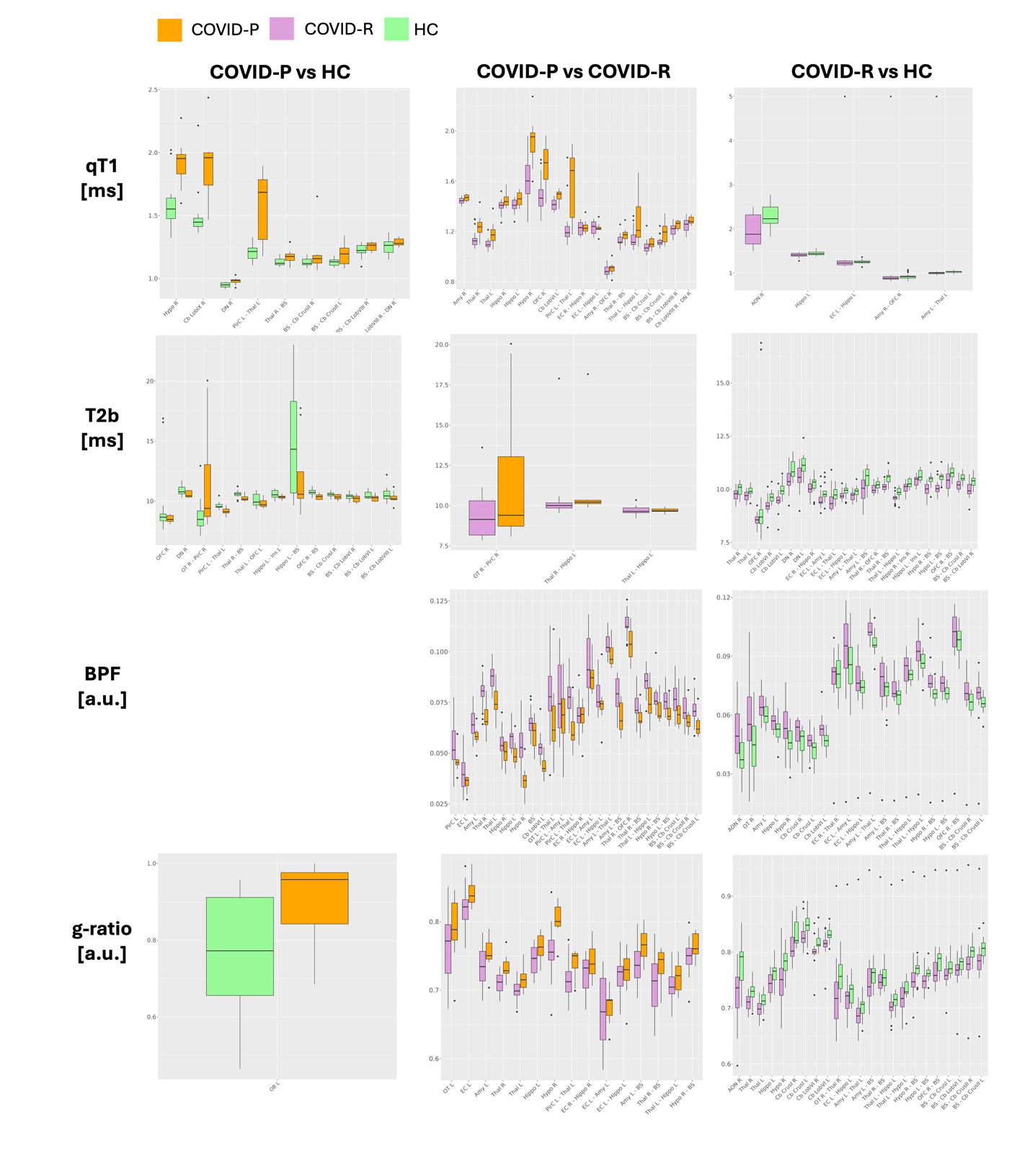


**Supplementary Fig.4:** **Boxplots of macromolecular-related metrics.** Boxplots of the macromolecular-related MRI metrics in the regions and tracts of the SoS atlas in HC (green), COVID-P (orange) and COVID-R (pink). Only significant differences (p<0.05) are reported for each comparison. The metrics are quantitative T1 (qT1), quantitative T2 of the bound pool component (T2b), bound-pool fraction (BPF), g-ratio.

HC: healthy controls, COVID-P: people with COVID-19 related persistent anosmia, COVID-R: people who recovered from COVID-19 related anosmia.


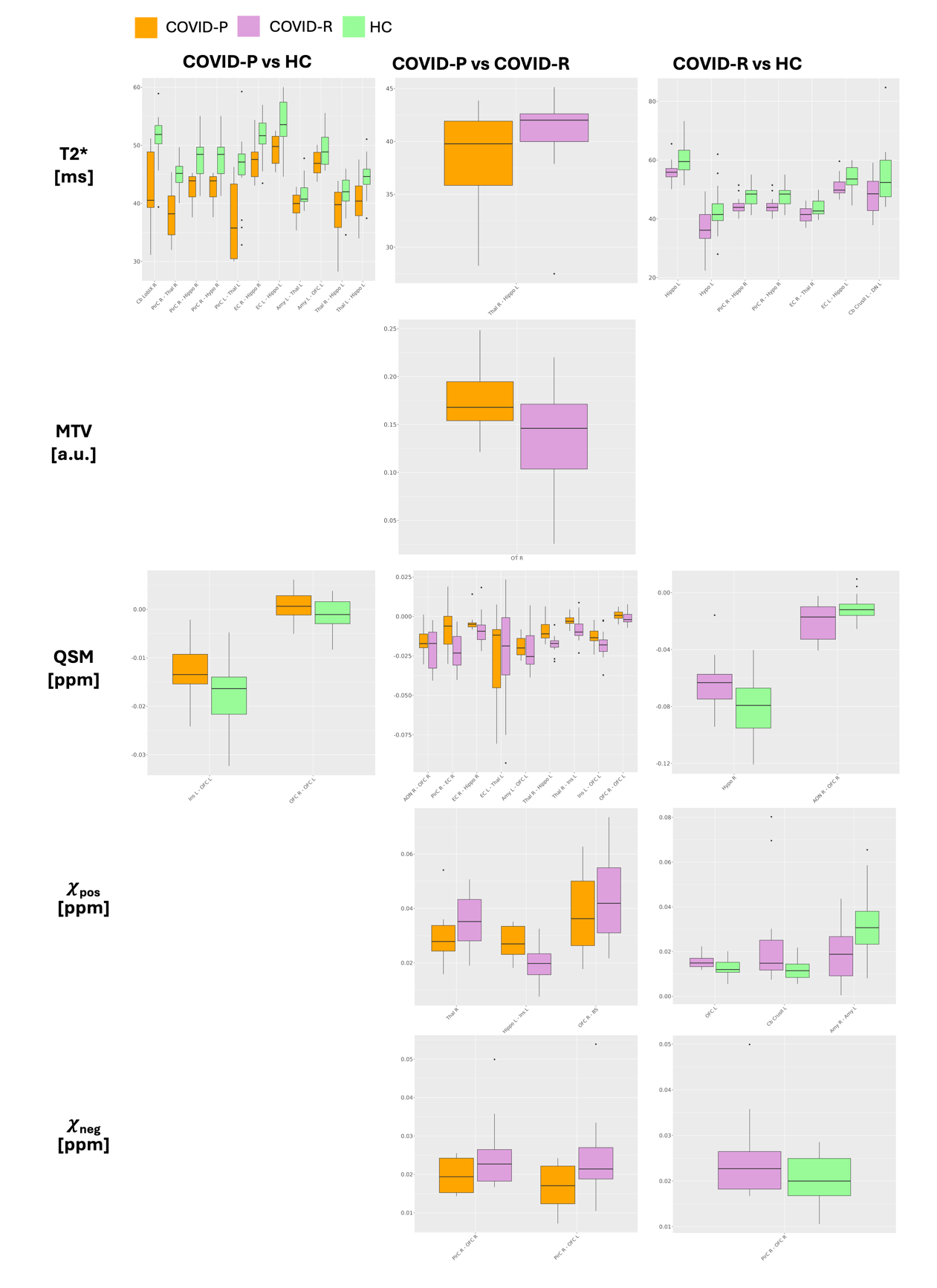


**Supplementary Fig.5:** **Boxplots of susceptibility-related metrics.** Boxplots of the susceptibility-related MRI metrics in the regions and tracts of the SoS atlas in HC (green), COVID-P (orange) and COVID-R (pink). Only significant differences (p<0.05) are reported for each comparison. The metrics are T2*, macromolecular tissue volume (MTV), quantitative susceptibility mapping (QSM), positive susceptibility (χ_pos_) and negative susceptibility (χ_neg_).

HC: healthy controls, COVID-P: people with COVID-19 related persistent anosmia, COVID-R: people who recovered from COVID-19 related anosmia.

**Supplementary Fig.6:** **Voxel-based analysis between groups in the SoS atlas.** The table on the left reports the grey matter regions (GM-ROI) SoS atlas while the table on the right reports the white matter tracts (WM-Tract) SoS atlas where a certain percentage of altered voxels was detected by the voxel-based analysis. For each comparison: **A**) COVID-P vs HC, **B)** COVID-P vs COVID-R, **C)** COVID-R vs HC, blue cells represent a decrease while red cells represent an increase of the MRI metrics in the first group. The analysed MRI maps are: grey matter (GM) and white matter (WM) probability maps, fractional anisotropy (FA), mean diffusivity (MD), intra-cellular volume fraction (v_intra_), isotropic volume fraction (v_iso_), quantitative T1 (qT1), quantitative T2 of the bound pool component (T2b), bound-pool fraction (BPF), g-ratio, macromolecular tissue volume (MTV), quantitative susceptibility mapping (QSM), positive susceptibility (χ_pos_) and negative susceptibility (χ_neg_).

HC: healthy controls, COVID-P: people with COVID-19 related persistent anosmia, COVID-R: people who recovered from COVID-19 related anosmia.


**Supplementary Fig.7:** **Voxel-based analysis between groups at whole brain level.** The table on the left shows the cortical (purple), subcortical (pink) and cerebellar (orange) regions while the table on the right shows the tracts where a certain percentage of altered voxels was detected by the voxel-based analysis in whole brain. For each comparison: **A**) COVID-P vs HC, **B)** COVID-P vs COVID-R, **C)** COVID-R vs HC, blue cells represent a decrease while red cells represent an increase of the MRI metrics in the first group. The analysed MRI maps are: grey matter (GM) and white matter (WM) probability maps, fractional anisotropy (FA), mean diffusivity (MD), intra-cellular volume fraction (v_intra_), isotropic volume fraction (v_iso_), quantitative T1 (qT1), bound-pool fraction (BPF), quantitative T2 of the bound pool component (T2b), g-ratio and quantitative susceptibility mapping (QSM), positive susceptibility (χ_pos_) and negative susceptibility (χ_neg_).

HC: healthy controls, COVID-P: people with COVID-19 related persistent anosmia, COVID-R: people who recovered from COVID-19 related anosmia.
