# Supplementary material for: The Sense of Smell (SoS) Atlas: Its Creation and First Application to Investigate COVID‐19 Related Anosmia With a Comprehensive Quantitative MRI Protocol
Source: J Magn Reson Imaging. 2025 Oct 3;63(2):574–93. doi: 10.1002/jmri.70128 (PMC12811004; doi:10.1002/jmri.70128)
Supplement: Supplementary file 1 — Data S1: jmri70128‐sup‐0001‐supinfo.zip. [file JMRI-63-574-s001.zip › Supplementary_results_clear.docx]

**Supplementary results**

**Anosmia related alterations in COVID-19: whole-brain**

When comparing COVID-P subjects to HC, the statistical differences were located mostly in the cerebellar regions and the intra HB tracts. COVID-P showed lower WM, v_intra_, BPF and χ_neg_, while higher values were observed in MD and qT1. In COVID-P, FA was generally lower, except in the cuneal cortex and left pallidum where it was higher. V_iso_ was generally lower, except in the right pallidum where it was higher. T2b was generally lower, except in the middle frontal gyrus, middle temporal gyrus (anterior division), inferior temporal gyrus (anterior division) and genu of corpus callosum, where it was higher. G-ratio was higher in middle temporal gyrus (anterior division and temporooccipital part) and inferior temporal gyrus (anterior division) while it was lower in the left superior cerebellar peduncle and in the right and left Cb LobI_IV. QSM was lower in superior temporal gyrus (posterior division) and right accumbens and higher in the inferior temporal gyrus (temporo-occipital part) and subcallosal cortex. All the variations in the regions were below 10%, except for the right accumbens in MD (10%). In the tracts, variations were more extensive across multiple metrics, especially in the right and left medial lemniscus: FA (respectively, 19% and 6%), MD (respectively, 49% and 5%), v_intra_ (respectively, 44% and 1%) and v_iso_ (respectively, 34% and 1%). The mainly affected tracts were the right and left inferior and superior cerebellar peduncle and cerebral peduncle, altered in FA, MD, v_intra_ and v_iso._

When comparing COVID-P to COVID-R subjects, the statistical differences were located mostly in the cortical regions and both intra forebrain and intra HB tracts. COVID-P showed lower values in GM, WM, FA, v_intra_, BPF and χ_pos_, while higher values were observed in MD, v_iso_, qT1, T2b, g-ratio and QSM. The mainly affected region was the left caudate in MD (24%) and v_iso_ (20%). The mainly affected tracts were the right medial lemniscus in MD (23%) and v_iso_ (10%), the left medial lemniscus in T2b (10%), the right inferior cerebellar peduncle in MD (17%) and v_iso_ (12%) and the right superior cerebellar peduncle in MD (7%) and FA (11%).

When comparing COVID-R subjects to HC, the statistical differences were located mostly in the cortical and cerebellar regions and intra HB tracts. COVID-R showed a lower WM, v_iso_, qT1, T2b and g-ratio, while higher values were observed in FA, v_intra_, BPF, χ_pos_ and χ_neg_. In COVID-R, QSM was lower in the tracts, while in the regions it was higher. T2b was the most affected map, indeed it has higher values in several regions, such as right Cb LobX (20%), right Cb LobV (10%), left Cb LobI_IV (12%), temporal occipital fusiform cortex (13%), lingual gyrus (12%) and parahippocampal gyrus (posterior division) (14%), and several tracts, such as middle cerebellar peduncle (20%), pontine crossing tract (19%), left inferior cerebellar peduncle (13%), left superior cerebellar peduncle (15%), left cerebral peduncle (13%) and right cingulum (12%). The left cerebral peduncle exhibited a 18% change in the χ_pos_, while right cingulum exhibited a 11% change in the g-ratio.
