# Supplementary material for: The Sense of Smell (SoS) Atlas: Its Creation and First Application to Investigate COVID‐19 Related Anosmia With a Comprehensive Quantitative MRI Protocol
Source: J Magn Reson Imaging. 2025 Oct 3;63(2):574–93. doi: 10.1002/jmri.70128 (PMC12811004; doi:10.1002/jmri.70128)
Supplement: Supplementary file 1 — Data S1: jmri70128‐sup‐0001‐supinfo.zip. [file JMRI-63-574-s001.zip › Supplementary_table-clean.docx]

|  | **Tract (R)** | **Not-ROI** | **Tract (L)** | **Not-ROI** |
| --- | --- | --- | --- | --- |
| removed | PirC R-Cb CrusII L |  |  |  |
|  | EC R-Amy L |  |  |  |
|  | EC R-Thal L |  | Thal R-EC L |  |
|  | Amy R-Hippo R |  | Amy L-Hippo L |  |
|  | Amy R-Hypo L |  |  |  |
|  | Amy R-Cb CrusI L |  | Amy L-Cb CrusI R |  |
|  | Amy R-Cb CrusII L |  | Amy L-Cb CrusII R |  |
|  | Thal R-Amy L |  |  |  |
|  | Thal R-Thal L |  |  |  |
|  | Thal R-Cb CrusI L |  | Thal L-Cb CrusI R |  |
|  | Thal R-Cb LobVI L |  | Thal L-Cb LobVI R |  |
|  | Thal R-Cb CrusII L |  | Thal L-Cb CrusII R |  |
|  | Thal R-Cb LobVIII L |  | Thal L-Cb LobVIII R |  |
|  | Thal R-Cb LobIX L |  | Thal L-Cb LobIX R |  |
|  | Hippo R-Amy L |  |  |  |
|  | Hippo R-OFC R |  | Hippo L-OFC L |  |
|  | Hypo R-Amy L |  |  |  |
|  | Hypo R-Thal L |  | Thal R-Hypo L |  |
|  | Hypo R-Hippo L |  |  |  |
|  | Hypo R-Cb CrusI L |  | Hypo L-Cb CrusI R |  |
|  | Hypo R-Cb CrusII L |  | Hypo L-Cb CrusII R |  |
|  | Hypo R-Cb LobIX L |  | Hypo L-Cb LobIX R |  |
|  | OFC R-Thal L |  | Thal R-OFC L |  |
|  | OFC R-Cb CrusI L |  |  |  |
|  | OFC R-Cb CrusII L |  | OFC L-Cb CrusII R |  |
| modified | AON R-OFC R | Commissures, Ins R |  |  |
|  | OT R-Amy R | BS and PirC R | OT L-Amy L | BS and PirC R |
|  | OT R-OFC L | Commissures, Ins L, cingulum gyrus, cortex R |  |  |
|  | PirC R-Thal R | Cingulate cortex, Occipital and frontal cortices R, Commissures | PirC L-Thal L | Cingulate cortex, Occipital and frontal cortices L, Commissures |
|  | PirC R-Hippo R | Cingulate cortex R, corpus callosum e BS |  |  |
|  | PirC R-OFC R | Corpus callosum, superior longitudinal fasciculus R, Ins R , limbic plane R, supra-Thal plane R | PirC L-OFC L | Corpus callosum, superior longitudinal fasciculus L, Ins L , limbic plane L, supra-Thal plane L |
|  | PirC R-BS | Cb R and L, Commissures, cingulate cortex and limbic plane R, frontal cortex R and Thal L | PirC L-BS | Cb R and L, Commissures, cingulate cortex and limbic plane L, and frontal cortex L |
|  | EC R-Amy R | superior longitudinal fasciculus R, limbic plane R, Hippo R, BS and fornix | EC L-Amy L | superior longitudinal fasciculus L, limbic plane L, Hippo L, Amy L, BS, fornix and corpus callosum |
|  | EC R-Thal R | Cingulate cortex, occipital lobe R, fornix, Amy R, Commissures, BS, and superior longitudinal fasciculus R | EC L-Thal L | Cingulate cortex, occipital lobe L, fornix, Amy L, Commissures, BS and superior longitudinal fasciculus L |
|  | EC R-Hippo R | Fornix, Amy R, Commissures, and occipital lobe R | EC L-Hippo L | Fornix, Amy L, Commissures, and occipital lobe L |
|  | EC R-Hypo R | BS, limbic plane R, piriform cortex R, and corpus callosum |  |  |
|  | EC R-OFC R | Frontal cortex R, BS, superior longitudinal fasciculus R, Ins R, and supra-Thal plane R | EC L-OFC L | Frontal cortex L, BS, superior longitudinal fasciculus L, Ins L, and supra-Thal L |
|  | EC R-BS | Commissures, cerebellum R and L, and cingulate cortex | EC L-BS | Commissures, cerebellum R and L, and cingulate cortex |
|  | Amy R-Amy L | Corpus callosum, BS, limbic plane R and L, cortices R and L |  |  |
|  | Amy R-Thal R | Hippo R, corpus callosum, BS, primary motor and somatosensory cortices R e supra-Thal plane R | Amy L-Thal L | Hippo L, corpus callosum, BS, primary motor and somatosensory cortices L, supra-Thal plane L and cingulate cortex R and L |
|  | Amy R-Hypo R | BS, fornix, supra-Thal plane R, and limbic plane R | Amy L-Hypo L | BS, fornix, supra-Thal plane L, and limbic plane L |
|  |  |  | Amy L-Ins L | Limbic plane L, supra-Thal plane L, parietal and occipital lobes L and Commissures |
|  | Amy R-OFC R | Cingulate cortex, Ins R, BS superior longitudinal fasciculus R, corpus callosum, supra-Thal plane R, limbic plane R, and frontal cortex R | Amy L-OFC L | Cingulate cortex, Ins L, BS, superior longitudinal fasciculus L, corpus callosum (body), Ins L, supra-Thal plane L, limbic plane L, frontal cortex L and Thal L |
|  | Amy R-BS | Fornix, Commissures, cerebellum R, supra-Thal plane R, occipital lobe R, Hippo R, and limbic plane R | Amy L-BS | Fornix, Commissures, cerebellum L, supra-Thal plane L, occipital lobe L, Hippo L, and limbic plane L |
|  | Thal R-Hippo R | Parietal lobe R, cingulate cortex, corpus callosum, BS, supra-Thal plane R, primary motor cortex and somatosensory cortex R | Thal L-Hippo L | Parietal lobe L, cingulate cortex, corpus callosum, BS, supra-Thal plane L, primary motor cortex and somatosensory cortex L |
|  | Thal R-Hypo R | supra-Thal plane R, limbic plane R, BS, prefrontal cortex R, cerebellum R, cortex R, and fornix | Thal L-Hypo L | supra-Thal plane L, limbic plane L, BS, prefrontal cortex L, cerebellum L, cortex L and fornix |
|  | Thal R-Ins R | Parietal and occipital lobes R, supra-Thal plane R, primary motor cortex R, superior longitudinal fasciculus R, and cingulate cortex | Thal L-Ins L | Parietal and occipital lobes L, supra-Thal plane L , Primary motor cortex L, superior longitudinal fasciculus L and cingulate corte |
|  | Thal R-OFC R | Commissures, BS, cingulate cortex, superior longitudinal fasciculus R, Hippo R, fornix, limbic plane R, supra-Thal plane R, frontal cortex R and Ins R | Thal L-OFC L | Commissures, BS, cingulate cortex, superior longitudinal fasciculus L, Hippo L, fornix, limbic plane L, supra-Thal plane L, frontal cortex L and Ins L |
|  | Thal R-BS | Cingulate cortex, cortices R and L, Hippo R, Commissures, cerebellum R and L, supra-Thal planes L and R | Thal L-BS | Cingulate cortex, cortices R and L, Hippo L, Commissures, cerebellum R and L, supra-Thal planes L and R |
|  | Hippo R-Hypo R | BS, fornix, limbic plane R, cingulate cortex, and supra-Thal plane R | Hippo L-Hypo L | BS, fornix, limbic plane L, cingulate cortex, and supra-Thal plane L |
|  | Hippo R-BS | Corpus callosum, primary motor cortex and somatosensory cortex R, parietal cortex R, supra-Thal plane R, cingulate cortex, occipital lobe R, cerebellum R and L | Hippo L-BS | Corpus callosum, primary motor cortex and somatosensory cortex R, parietal cortex L, supra-Thal plane L, cingulate cortex, occipital lobe L, cerebellum R and L, Thal L |
|  | Hypo L-OFC L | BS, Ins L, Commissures, cingulate cortex L, supra-Thal plane L, frontal cortex L, and limbic plane L |  |  |
|  | Hypo R-BS | Commissures, cerebellum R and L, fornix, and supra-Thal plane R | Hypo L-BS | Commissures, cerebellum R and L, fornix, and supra-Thal plane L and Thal L |
|  | Ins R-BS | Corpus callosum, primary motor cortex R, and somatosensory cortex R | Ins L-BS | Corpus callosum, primary motor cortex L, and somatosensory cortex L |
|  | OFC R-OFC L | Ins R and L, frontal cortex R and L |  |  |
|  | OFC R-BS | Commissures, cerebellum R and L, cingulate cortex R, supra-Thal plane R, Ins R, frontal cortex R, and limbic plane R | OFC L-BS | Commissures, cerebellum R and L, cingulate cortex L, supra-Thal plane L, Ins L, frontal cortex L, and limbic plane L |
|  | BS-Cb CrusI R | Cerebellum L, Cb CrusII R, cortices R and L, Cb LobIX R | BS-Cb CrusI L | Cerebellum R, Cb Crus II L, cortices R and L and Cb LobIX L |
|  | BS-Cb LobVI R | Cerebellum L, Cb LobIX R, Cb CrusI L, Cb CrusII L, cortex L | BS-Cb LobVI L | Cerebellum R, Cereb IX L, crus I and II L, cortex L |
|  | BS-Cb CrusII R | Cerebellum L, Cb LobVIII R and Cb LobIX R | BS-Cb CrusII L | Cerebellum R, Cb LobVIII L and Cb LobIX L |
|  | BS-Cb LobVIII R | Cerebellum L and Cb LobIX R | BS-Cb LobVIII L | Cerebellum R and Cb LobIX L |
|  | BS-Cb LobIX R | Cerebellum L and Cb LobVIII R | BS-Cb LobIX L | Cerebellum R, Thal L, and Cb LobVIII L |
|  | BS-DN R | Cb LobVIII R, Cb LobIX R and cerebellum L | BS-DN L | Cb LobIX L and cerebellum R |
|  | Cb CrusI R-DN R | Cb LobVIII R, Cb LobIX R, BS and eroded Cb Crus II R | Cb CrusI L-DN L | Cb LobVIII L, BS and Cb Crus II L |
|  | Cb LobVI R-DN R | Cb CrusI R, Cb CrusII R, Cb LobVIII R, Cb LobIX R, BS | Cb LobVI L-DN L | Cb CrusI L, Cb CrusII L, Cb LobVIII L, BS |
|  | Cb CrusII R-DN R | BS and Cb LobVIII R | Cb CrusII L-DN L | BS and Cb LobVIII L |
|  | Cb LobVIII R-DN R | BS and Cb LobIX R | Cb LobVIII L-DN L | BS and Cb LobIX L |
|  | Cb LobIX R-DN R | Cb LobVIII R, Cb CrusII R, and BS | Cb LobIX L-DN L | Cb LobVIII L, Cb CrusII L, and BS |
| maintained | OT R-PirC R |  | OT L-PirC L |  |
|  | OT R-Thal R |  | OT L-Thal L |  |
|  | OT R-Hypo R |  | OT L-Hypo L |  |
|  | OT R-BS L |  |  |  |
|  | PirC R-EC R |  |  |  |
|  | PirC R-Amy R |  | PirC L-Amy L |  |
|  | PirC R-Thal L |  |  |  |
|  | PirC R-Hypo R |  | PirC L-Hypo L |  |
|  | Amy R-Thal L |  |  |  |
|  | Thal R-Hippo L |  | Hippo R-Thal L |  |
|  | Thal R-Ins L |  | Ins R-Thal L |  |
|  | Hippo L-Ins L |  |  |  |
|  | Hypo R-Hypo L |  |  |  |
|  | Ins R-OFC R |  | Ins L-OFC L |  |

**Supplementary Table 1: Curation process of the tracts of the olfactory circuit.** This table summarizes all the tracts and the curation process. First and third columns list the tracts, where symmetric tracts are reported in the same row. In the adjacent columns, the not-ROIs that have been used to curate the tracts are presented. Removed: tracts that have been entirely eliminated; modified: tracts that have been curated through not-ROI process, i.e., only certain streamlines have been removed; maintained: tracts that did not need any curation. OB: Olfactory Bulbs, AON: Anterior Olfactory Nucleus, OT: Olfactory Tubercle, PirC: Piriform Cortex, EC: Entorhinal Cortex, Amy: Amygdala, Thal: Thalamus, Hippo: Hippocampus, Hypo: Hypothalamus, Ins: Insula, OFC: Orbitofrontal Cortex, BS: Brain Stem, Cb Crus I: Cereb Crus I, Cb Lob VI: Cereb Lobule VI, Cb Crus II: Cereb Crus II, Cb Lob VIII: Cereb Lobule VIII, Cb Lob IX: Cereb Lobule IX, DN: Dentate.

**Supplementary Table 2: Region-based analysis results.** For each of the 111 regions and 13 metrics, the table reports the mean ± standard deviation for COVID-P, COVID-R, and HC, with the p-values and intercepts of the comparisons. Specifically, p1/i1 corresponds to the p-value/intercept for the comparison COVID-P vs. HC, p2/i2 for COVID-P vs COVID-R, and p3/i3 for COVID-R vs HC. To maintain consistency and avoid reducing the sample size, statistical analyses were not performed for regions where at least one subject had a missing value, resulting in NaN for p/i. Missing values mainly reflected very small regions that were not retained after atlas registration at lower resolution, and/or from the exclusion of a large number of outliers following the interquartile range (IQR) rule.

HC: healthy controls, COVID-P: people with COVID-19 related persistent anosmia, COVID-R: people who recovered from COVID-19 related anosmia.

See file excel: *Supplementary_Table2.xlsx*

**Supplementary Table 3: Voxel-based analysis results.** For each region and each metric, the table reports only the significant results. For each comparison, the mean ± standard deviation of the metric and the T-score in the altered voxels are reported. In each cell, the first mean ± standard deviation corresponds to the first group of the comparison, the second mean ± standard deviation corresponds to the second group, and the T-score for that comparison is reported last.

HC: healthy controls, COVID-P: people with COVID-19 related persistent anosmia, COVID-R: people who recovered from COVID-19 related anosmia.

See file excel: *Supplementary_Table3.xlsx*

| **Region-based** | | COVID-P vs HC | | COVID-P vs COVID-R | | COVID-R vs HC | |
| --- | --- | --- | --- | --- | --- | --- | --- |
|  |  | GM-ROI | WM-Tract | GM-ROI | WM-Tract | GM-ROI | WM-Tract |
| **Localization** | | | | | | | |
| POC | | 12% | 15% | 19% | 25% | 26% | 23% |
| SOC | | 20% | 37% | 58% | 44% | 40% | 46% |
| HB | | 68% | 48% | 23% | 31% | 34% | 31% |
| **Biophysical meaning** | | | | | | | |
| Axonal integrity | FA | 4% | 12% | 9% | 13% | 6% | 2% |
|  | v_intra_ | 16% | 16% | 5% | 7% | 0% | 0% |
| Inflammation | MD | 20% | 10% | 12% | 5% | 2% | 2% |
|  | v_iso_ | 28% | 11% | 12% | 9% | 2% | 0% |
|  | qT1 | 12% | 11% | 19% | 13% | 6% | 5% |
| Macromolecular density and myelin | T2b | 12% | 19% | 0% | 4% | 20% | 30% |
|  | MTV | 0% | 0% | 2% | 0% | 0% | 0% |
|  | BPF | 0% | 0% | 23% | 21% | 23% | 23% |
|  | $\chi$_neg_ | 0% | 0% | 0% | 3% | 0% | 2% |
|  | g-ratio | 4% | 0% | 16% | 11% | 26% | 25% |
| Iron | T2* | 4% | 18% | 0% | 1% | 6% | 9% |
|  | $\chi$_pos_ | 0% | 0% | 2% | 2% | 6% | 1% |
| Iron and myelin | QSM | 0% | 3% | 0% | 11% | 3% | 1% |

**Supplementary Table 4**: The table summarizes the alterations of the region-based analysis, including their localization and the biophysical meaning, for each group comparison. All the alterations are reported as percentages and are divided in GM-ROI and WM-Tract SoS atlas. The localization shows how the alterations are divided into the primary olfactory cortex (POC), the second olfactory cortex (SOC) and the hindbrain (HB). The biophysical meaning shows how the alterations are distributed across each MRI-multimodal map.

| **Voxel-based** | | COVID-P vs HC | | COVID-P vs COVID-R | | COVID-R vs HC | |
| --- | --- | --- | --- | --- | --- | --- | --- |
|  |  | GM-ROI | WM-Tract | GM-ROI | WM-Tract | GM-ROI | WM-Tract |
| **Localization** | | | | | | | |
| POC | | 7% | 6% | 17% | 15% | 26% | 21% |
| SOC | | 17% | 61% | 28% | 74% | 16% | 47% |
| HB | | 76% | 33% | 55% | 11% | 58% | 32% |
| **Biophysical meaning** | | | | | | | |
| Axonal integrity | FA | 11% | 0% | 12% | 4% | 0% | 0% |
|  | v_intra_ | 15% | 17% | 15% | 0% | 0% | 0% |
|  | WM | 7% | 25% | 0% | 0% | 11% | 8% |
| Inflammation | MD | 22% | 0% | 11% | 0% | 0% | 0% |
|  | v_iso_ | 14% | 0% | 12% | 0% | 0% | 0% |
|  | qT1 | 21% | 8% | 12% | 5% | 14% | 8% |
|  | GM | 8% | 0% | 5% | 10% | 11% | 0% |
| Macromolecular density and myelin | T2b | 0% | 0% | 7% | 5% | 22% | 14% |
|  | MTV | 0% | 0% | 0% | 0% | 0% | 0% |
|  | BPF | 0% | 25% | 15% | 8% | 15% | 30% |
|  | $\chi$_neg_ | 0% | 0% | 2% | 0% | 6% | 0% |
|  | g-ratio | 0% | 17% | 7% | 10% | 13% | 22% |
| Iron | T2* | 0% | 0% | 0% | 0% | 0% | 0% |
|  | $\chi$_pos_ | 0% | 0% | 0% | 39% | 8% | 6% |
| Iron and myelin | QSM | 2% | 8% | 2% | 19% | 0% | 12% |

**Supplementary Table 5**: The table summarizes the alterations of the voxel-based analysis, including their localization and the biophysical meaning, for each group comparison. All the alterations are reported as percentages and are divided in GM-ROI and WM-Tract SoS atlas. The localization shows how the alterations are divided into the primary olfactory cortex (POC), the second olfactory cortex (SOC) and the hindbrain (HB). The biophysical meaning shows how the alterations are distributed across each MRI-multimodal map.

| **Whole brain** | | COVID-P vs HC | | COVID-P vs COVID-R | | | COVID-R vs HC | | | |
| --- | --- | --- | --- | --- | --- | --- | --- | --- | --- | --- |
| **Localization** | | | | | | | | | |  |
| GM | | | | | | | | | |  |
| Cortex | | 34% | | 60% | | | 51% | | |  |
| Subcortex | | 11% | | 20% | | | 5% | | |  |
| Cerebellum | | 55% | | 20% | | | 44% | | |  |
| WM | | | | | | | | | |  |
| Forebrain | | 11% | | 34% | | | 38% | | |  |
| HB | | 89% | | 66% | | | 62% | | |  |
| **Biophysical meaning** | | | | | | | | | |  |
|  | | GM | WM | | GM | WM | | GM | WM |  |
| Axonal integrity | FA | 14% | 27% | | 10% | 15% | | 0% | 2% |  |
|  | v_intra_ | 6% | 16% | | 5% | 5% | | 3% | 2% |  |
|  | WM | 8% | 0% | | 6% | 0% | | 12% | 0% |  |
| Inflammation | MD | 9% | 18% | | 12% | 23% | | 0% | 0% |  |
|  | v_iso_ | 12% | 14% | | 10% | 13% | | 3% | 0% |  |
|  | qT1 | 4% | 7% | | 8% | 0% | | 11% | 0% |  |
|  | GM | 0% | 0% | | 10% | 10% | | 3% | 5% |  |
| Macromolecular density and myelin | T2b | 16% | 11% | | 8% | 13% | | 24% | 22% |  |
|  | MTV | 0% | 0% | | 0% | 0% | | 0% | 0% |  |
|  | BPF | 7% | 0% | | 8% | 6% | | 9% | 11% |  |
|  | $\chi$_neg_ | 0% | 1% | | 0% | 0% | | 3% | 0% |  |
|  | g-ratio | 16% | 6% | | 6% | 7% | | 9% | 10% |  |
| Iron | T2* | 0% | 0% | | 0% | 0% | | 0% | 0% |  |
|  | $\chi$_pos_ | 0% | 0% | | 7% | 0% | | 6% | 41% |  |
| Iron and myelin | QSM | 8% | 0% | | 10% | 8% | | 17% | 7% |  |

**Supplementary Table 6**: The table summarizes the alterations of the voxel-based analysis at whole brain level, including their localization and the biophysical meaning, for each group comparison. All the alterations are reported as percentages and are divided in gray matter (GM) and white matter (WM). The localization shows how the alterations are divided into the cortex, subcortex and cerebellum (GM) and forebrain and hindbrain (WM). The biophysical meaning shows how the alterations are distributed across each MRI-multimodal map.
